# Associations Between Colonic Motor Patterns and Autonomic Nervous System Activity Assessed by High-Resolution Manometry and Concurrent Heart Rate Variability

**DOI:** 10.3389/fnins.2019.01447

**Published:** 2020-01-23

**Authors:** Yuhong Yuan, M. Khawar Ali, Karen J. Mathewson, Kartik Sharma, Mahi Faiyaz, Wei Tan, Sean P. Parsons, Kailai K. Zhang, Natalija Milkova, Lijun Liu, Elyanne Ratcliffe, David Armstrong, Louis A. Schmidt, Ji-Hong Chen, Jan D. Huizinga

**Affiliations:** ^1^Department of Gastroenterology, Sun Yat-sen Memorial Hospital, Sun Yat-sen University, Guangzhou, China; ^2^Department of Medicine, Division of Gastroenterology, Farncombe Family Digestive Health Research Institute, McMaster University, Hamilton, ON, Canada; ^3^School of Biomedical Engineering, McMaster University, Hamilton, ON, Canada; ^4^Department of Psychology, Neuroscience, and Behaviour, McMaster University, Hamilton, ON, Canada; ^5^Department of Gastroenterology, Renmin Hospital of Wuhan University, Wuhan, China; ^6^Department of Pediatrics, Farncombe Family Digestive Health Research Institute, McMaster University, Hamilton, ON, Canada

**Keywords:** RSA, PEP, baevsky stress index, colonic motility, autonomic nervous system, parasympathetic, sympathetic, high-amplitude pressure waves

## Abstract

Abnormal colonic motility may be associated with dysfunction of the autonomic nervous system (ANS). Our aim was to evaluate if associations between colonic motor patterns and autonomic neural activity could be demonstrated by assessing changes in heart rate variability (HRV) in healthy volunteers. A total of 145 colonic motor patterns were assessed in 11 healthy volunteers by High-Resolution Colonic Manometry (HRCM) using an 84-channel water-perfused catheter. Motor patterns were evoked by balloon distention, a meal and luminal bisacodyl. The electrocardiogram (ECG) and cardiac impedance were assessed during colonic manometry. Respiratory sinus arrhythmia (RSA) and root mean square of successive differences of beat-to-beat intervals (RMSSD) served as measures of parasympathetic reactivity while the Baevsky’s Stress Index (SI) and the pre-ejection period (PEP) were used as measures of sympathetic reactivity. Taking all motor patterns into account, our data show that colonic motor patterns are accompanied by increased parasympathetic activity and decreased sympathetic activity that may occur without eliciting a significant change in heart rate. Motor Complexes (more than one motor pattern occurring in close proximity), High-Amplitude Propagating Pressure Waves followed by Simultaneous Pressure Waves (HAPW-SPWs) and HAPWs without SPWs are all associated with an increase in RSA and a decrease in SI. Hence RSA and SI may best reflect autonomic activity in the colon during these motor patterns as compared to RMSSD and PEP. SI and PEP do not measure identical sympathetic reactivity. The SPW, which is a very low amplitude pressure wave, did not significantly change the autonomic measures employed here. In conclusion, colonic motor patterns are associated with activity in the ANS which is reflected in autonomic measures of heart rate variability. These autonomic measures may serve as proxies for autonomic neural dysfunction in patients with colonic dysmotility.

## Introduction

Colonic motility is regulated by a multitude of control systems. The colonic musculature receives rhythmic depolarization from the colonic pacemaker networks of interstitial cells of Cajal (ICC) and on-demand depolarization from the enteric nervous system (Furness, 2012; Huizinga, 2018). Motor patterns are generated in response to stimuli that are mediated by sensory and motor neurons, intrinsic and extrinsic to the musculature (Bharucha et al., 1993, 2008; Brierley et al., 2004; Costa and Brookes, 2008; Wang et al., 2009; Knowles et al., 2013; Callaghan et al., 2018). The extrinsic nervous system facilitates brain – colon communication, which plays a critical role in many aspects of colonic motility such as the defecation reflex (Szurszewski et al., 2002; Tache, 2003; Brookes et al., 2016).

The defecation reflex starts with a sensation of urgency mediated by rectal stimulation followed by sacral sensory nerve activation. This impulse initiates activity in the sacral defecation center, which sends signals to brain stem areas and the frontal cortex, which in turn activate motor nerves from the ANS and the enteric nervous system to produce a bowel movement (Furness, 2012; Spencer et al., 2016). The essential role of the ANS is demonstrated by the fact that the defecation reflex is lost when sacral parasympathetic activity is absent due to spinal injury (Devroede and Lamarche, 1974). Similarly, children with bowel dysfunction show significantly lower parasympathetic reactivity (Fazeli et al., 2016). Dramatic reduction in colonic motor activity may also occur when sympathetic activation is prolonged during stress (McIntyre and Thompson, 1992). Attempts have been made to gain insight into brain-gut communication through analysis of heart rate variability (HRV); Irritable Bowel Syndrome (IBS) patients with constipation appear to have decreased levels of resting parasympathetic activity or increased resting sympathetic activity, suggesting that parasympathetic withdrawal with or without sympathetic activation could be an important contributing factor to constipation (Mazurak et al., 2012; Polster et al., 2018).

Heart rate is the net outcome of the intrinsic sinus node pacemaker activity that is influenced by sympathetic and parasympathetic innervation of the heart (Draghici and Taylor, 2016). HRV analysis is based on the peak R-R interval time series obtained from the ECG. HRV reflects physiological variation in heart rate caused by its autonomic innervation, but heart rate regulation may be influenced by events that are only indirectly related to physiological regulation of the heart rate; HRV may reflect ANS activity more generally, including associations with the gut (Mathewson et al., 2014; Bonaz et al., 2016; Draghici and Taylor, 2016; Ernst, 2017). The NTS in the brain stem plays a key role in this since it is involved in regulation of both heart rate and gut motility (Browning and Travagli, 2014; Babic et al., 2015).

Parasympathetic regulatory activity is typically represented by the intensity of a high frequency component (0.15–0.40 Hz) of HRV that fluctuates with the phase of respiration (Bonaz et al., 2016), known as RSA. Higher levels of resting RSA in healthy individuals are associated with the ability to adapt quickly to internal and external changes in the environment (Beauchaine and Thayer, 2015). An additional measure of parasympathetic activity is the root mean square of successive differences (RMSSD) between adjacent beat-to-beat intervals, which is calculated in the time domain.

The PEP, refers to the latency of the ejection of blood into the aorta, an index that correlates with ventricular contractility (Quigley and Stifter, 2006) and measured by impedance cardiography. Lower PEP values reflect greater contractility (increased sympathetic activation), whereas higher values reflect lower contractility (decreased sympathetic activation). In addition, we employed the Baevsky’s Stress Index (SI), calculated from the main characteristics of the histogram of beat-to-beat intervals, the mode (the RR interval value repeating the most in the signal), the amplitude of the mode, and the range in variation. SI values increase with increases in sympathetic nervous system activity (Baevsky and Chernikova, 2017).

Autonomic measures are influenced by exercise, sleep, postprandial activity, smoking, alcohol, and coffee consumption in normal healthy individuals (Ernst, 2017). Other factors, such as gender, age, breathing frequency, body mass index, blood pressure, negative feelings (eg., depression, stress, pain), hormonal activity, medications, even family history and genetic background may influence HRV (Parati and Di Rienzo, 2003), indicating that HRV reflects more than cardiac health.

The objective of the present study was to test the hypothesis that activity in the ANS associated with colonic motor patterns can be observed as changes in HRV and impedance measurements. If so, these measures may provide a way to assess autonomic dysfunction in patients with colonic dysmotility. To this end, we conducted HRCM while simultaneously recording the ECG and cardiac impedance measures.

## Materials and Methods

### Participants

Eleven healthy participants were recruited by local advertisement (Table 1). Participants with any history suggestive of current or prior cardiovascular or gastrointestinal disease were excluded. Participants were not taking drugs that might affect cardiac or gastrointestinal function. Ethics approvals were obtained from McMaster University, the Hamilton Integrated Research Ethics Board, and written consent was obtained from all participants. The entire study was conducted at McMaster University.

**TABLE 1:** Participant characteristics (*n* = 11).

	**Mean ± SD**	**Range**
Gender: male: 7 (64%); female 4 (36%)		
Age (yr)	32.211.3	21—54
BMI (kg/m^2^)	25.73.8	19.6-31.5
Baseline autonomic function (supine)		
RSA (ln ms^2^)	6.70.97	5.47–8.68
RMSSD (ms)	57.930.46	27.9–134.3
PEP (ms)	121.6417.17	88–145.33
SI (ms^–2^)	32.8523.09	8.03–87.01
HR (bpm)	63.788.9	54.0–81.3

### Methodologies and Nomenclature

High-Resolution Colonic Manometry was performed using an 84-sensor water perfused catheter that detected luminal pressures at 1 cm intervals from the proximal colon to the anal sphincter (Chen et al., 2017). The catheter was custom-made by Mui Scientific (Mississauga, ON, Canada) and the acquisition hardware was custom made by Medical Measurement Systems (Laborie, Toronto, ON, Canada). The manometric analysis was aided by customized Image-J and MATLAB software. This study reports on the ECG and impedance data ^30^ obtained simultaneously with the HRCM data from the entire colon (Chen et al., 2018).

**FIGURE 1:**
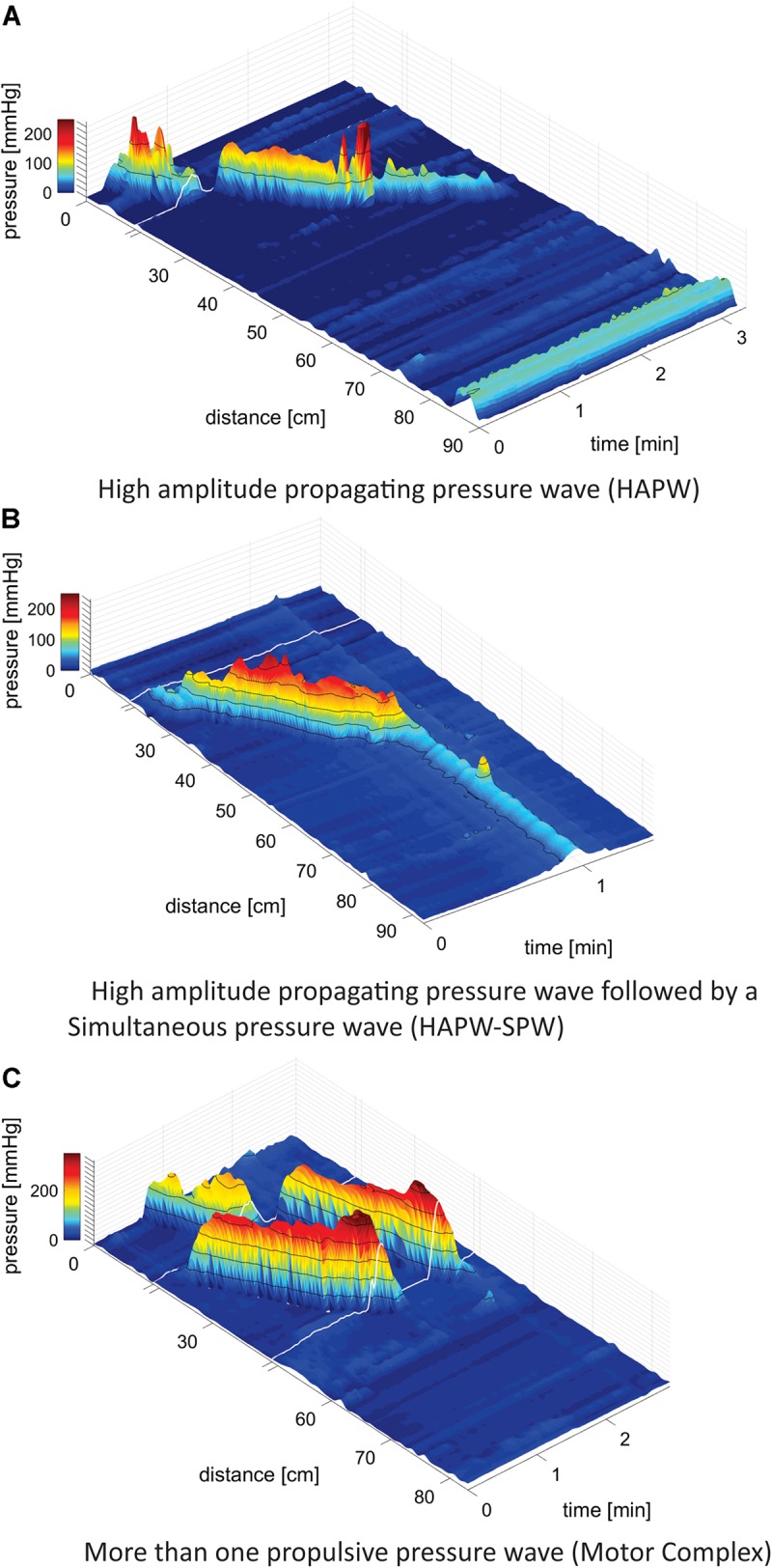
Motor patterns. **(A)** High-Amplitude Propagating Pressure Waves (HAPWs) occurring as a single isolated event. White line indicates the presence of a 10 cm long balloon in the catheter where no data could be collected. The anal sphincter pressure is seen at 86 cm. 0 cm is in the proximal colon. **(B)** A HAPW followed by a simultaneous pressure wave (SPW) referred to as HAPW-SPW. White line indicates the presence of a 10 cm long balloon in the catheter where no data could be collected. The catheter was fully inside the colon such that the anal sphincter activity was not recorded. The HAPW started distal to the balloon. **(C)** Motor Complexes, defined as two or more distinct propulsive motor patterns that followed each other closely so that HRV changes could not be assessed for the individual patterns. In this catheter, two balloons were present at the white lines so that two sections of 10 cm did not have pressure sensors. No anal activity was recorded.

The following motor patterns were analyzed in conjunction with the autonomic measures:

• High-Amplitude Propagating Pressure Waves (HAPWs), occurring as single isolated events (Bharucha, 2012; Figure 1A).

• High-Amplitude Propagating Pressure Waves followed by SPWs referred to as HAPW-SPWs (Chen et al., 2017, 2018; Figure 1B).
• Motor Complexes (MCs): when two or more distinct motor patterns followed each other closely so that HRV changes could not be assessed for the individual patterns, e.g., two or more HAPWs (Figure 1C), or 1 HAPW and 1 HAPW-SPW etc.
• Simultaneous Pressure Waves (SPWs), most often pancolonic, occurring as single isolated events (Chen et al., 2017, 2018).

ECG and impedance cardiography were recorded from seven electrodes on the subject’s torso. Three electrodes formed a modified Lead II configuration for ECG recording. Four more electrodes were used in a standard tetrapolar electrode configuration for impedance recording, where two electrodes supplied a constant current source and two electrodes registered the changes in the transfer impedance (reflecting changes in activity of the sympathetic nervous system). Signals were recorded using a MindWare impedance cardio GSC monitor. HRV and impedances were analyzed by MindWare HRV 3.1 and IMP 3.1 software (MindWare Technologies Ltd., Gahanna, OH, United States). To assess general parasympathetic reactivity, we recorded changes in RSA and RMSSD in response to changes in body position during a resting baseline and posture change. Similarly, sympathetic reactivity was assessed by changes in PEP and in the Baevsky’s stress index (SI).

### General Autonomic Testing Prior to HRCM

#### Visit 1

Resting autonomic measures appear highly stable within an individual (Schmidt et al., 2012). Therefore, all volunteers underwent autonomic testing between 1 and 4 weeks prior to HRCM testing. Participants were accommodated in a quiet room with normal lighting and temperature. They had been asked to refrain from smoking, caffeine, alcohol and heavy eating in the 2 h before testing. After lying quietly in supine position for at least 10 min, ECG and impedance were measured for a 6 min rest period in the supine position. To test general autonomic reactivity, the ECG was monitored while the subject was sitting on the edge of the bed, standing still, and while walking on the spot, each for 6 min (Jáuregui-Renaud et al., 2001).

### HRCM, HRV and Impedance Recording

#### Visit 2

In addition to the general autonomic testing, all participants underwent simultaneous HRCM, ECG, and impedance recording. Bowel preparation with a high-volume polyethylene glycol electrolyte solution (Peglyte 280g, Pendopharm, Montreal, QC, Canada) was performed starting at 4 PM the day before HRCM (Chen et al., 2018). No other stimuli were used for bowel cleaning. The catheter was placed into the colon via colonoscopy and attached in the proximal colon mucosa with a clip. In six subjects, two balloons were part of the catheter, placed between sensors 10 and 11 and 40 and 41. In five subjects only a proximal balloon was part of the catheter placed between sensors 10 and 11. The balloons take up 10 cm space in which no pressure recordings can be made; these data omissions are indicated by a white line in the figures showing three-dimensional color manometry graphs. Manometric and autonomic recordings were performed during 90 min of baseline rest after colonoscopy, followed by 20 min of proximal balloon distention, 20 min of rectal balloon distention using a standard anorectal manometry balloon assembly, 90 min following intake of a meal, consisting of organic yogurt fortified by organic milk fat to make it 1000 kcal (Mapleton Organic, Ontario, Canada), and 45 min after administration of rectal bisacodyl. Participants were supine during all recordings except during the actual intake of the meal.

Autonomic reactivity was examined in the following conditions. (1) Baseline rest: supine autonomic activity obtained during general autonomic testing. This measure was taken to evaluate whether changes in HRV due to motor patterns were related to the subject’s “basal” autonomic activity. (2) Autonomic reactivity in response to body position changes from supine to sitting, then standing and walking. (3) Autonomic reactivity to colonic motor patterns by comparing three conditions: (a) pre-motor-pattern, the 2 min prior to the occurrence of the motor pattern. (b) the motor-pattern: autonomic activity during the entire period in which the motor pattern was present. (c) the recovery period, measured in the first 2 min immediately after the motor pattern.

### Statistical Analyses

All data sets were assessed for normal distribution by applying the Shapiro–Wilk normality test (Ghasemi and Zahediasl, 2012) using GraphPad Prism version 8, RSA, RMSSD, PEP, SI, and HR values were assessed or compared during different postures and in response to motor patterns. Data were expressed as mean ± S.E.M. *N* = number of motor patterns. *n* = number of subjects.

### General Autonomic Testing

At the first visit, general autonomic activity was assessed across postural conditions (supine, sitting, standing, walking) for each autonomic measure (Jáuregui-Renaud et al., 2001). All data sets were normally distributed except SI data. Normally distributed data sets were analyzed using one-way ANOVA with follow up multiple comparison Bonferroni test. The SI data were assessed using the non-parametric Friedman test followed by Dunn’s multiple comparison test.

### HRCM and HRV

At a second visit, 1–4 weeks later, A HRCM assessment was performed while simultaneously measuring heart rate and impedance. Then HRV parameters were calculated associated with the motor patterns. To be eligible for analysis, each motor pattern needed to be preceded by a 2 min baseline period and followed by a 2 min recovery period, both without movement artifact or other motor patterns. The Shapiro–Wilk normality test indicated that the data sets were not normally distributed.

First, all the motor patterns in all subjects were assessed as a single group to measure the changes in HRV parameters. Second, each category of motor patterns was assessed separately. First, the question was answered whether or not the occurrence of a motor pattern was associated with a change in any of the HRV parameters using the non-parametric Wilcoxon matched pairs signed rank test, comparing 2 min before to time during motor pattern. The data were averaged per individual hence *n* = 11, indicated as W-M (Wilcoxon-Mean) in the text when *p* values are noted.

Second, the question was answered whether or not there was recovery from a change in any of the HRV parameters within the 2 min following the motor patters. This was a comparison between three data sets (before – during – after) and the data sets were not normally distributed and were paired. Hence, the Friedman test was applied followed by the Dunn’s multiple comparisons test. For each of the analyses, values of the motor patterns within one subject were averaged and considered an *n* = 1, indicated by F-D-M (Friedman-Dunn-Mean).

To provide a complete data set for all individual motor patterns in each category, graphs were presented with % change to emphasize the strength of the autonomic reactivity and its recovery. Within each category, the data were subjected to the Friedman test followed by Dunn’s multiple comparisons test, based on the number of motor patterns (N), indicated by (F-D).

### Correlation Between General Autonomic Testing and Autonomic Reactivity During Motor Patterns

Anticipating that patients with dysmotility might have reduced autonomic reactivity associated with motor patterns, and that this might be related to low general autonomic reactivity, we tested in the present study on healthy subjects whether or not any of the motor pattern associated changes in autonomic activity (comparing before and during motor patterns), was associated with two parameters of the general autonomic test, namely the supine value and the change from supine to standing. This was done using the Pearson Correlation Coefficient, two tailed test. Results with R^2^ > 0.5 and *p* < 0.05 were considered significantly correlated. In the text, data related to correlations between general autonomic testing and autonomic reactivity during motor patterns supine HRV and changes in HRV parameters only show significant changes, while the results with no correlation (*R*^2^ < 0.05 and/or p > 0.05) are provided in Supplementary Figures.

## Results

### General Autonomic Reactivity

**FIGURE 2:**
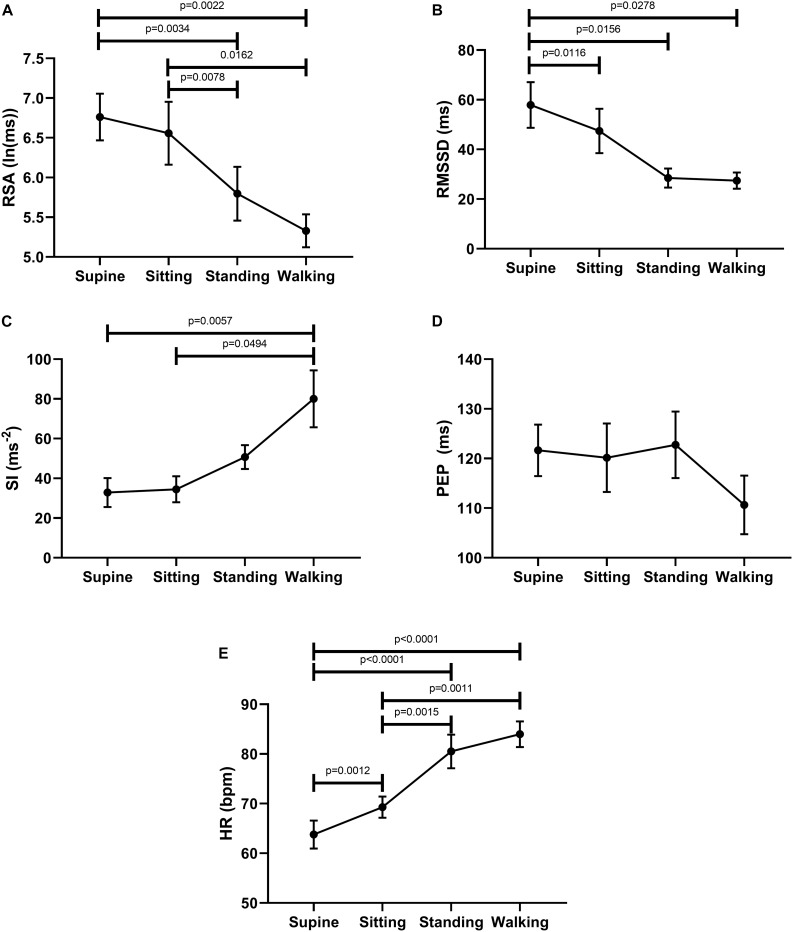
Changes in autonomic activity in response to posture changes (*n* = 11), Significance was assessed by one-way ANOVA with repeated measures and Bonferroni correction for **(A,B,D,E)**. Friedman test followed by Dunn’s multiple comparison tests was used to assess significance for **(C)**. **(A)** RSA declined upon standing and walking compared to the supine baseline position. **(B)** RMSSD declined upon standing and walking compared to the supine baseline position. **(C)** SI increased upon standing and walking compared to baseline position. **(D)** PEP decreased significantly only during walking. **(E)** Heart rate increases upon standing and walking compared to the supine baseline position.

The ability of the ANS to facilitate the generation of colonic motor patterns, may be related to a person’s general autonomic reactivity (Shaffer and Ginsberg, 2017), which was therefore measured. General autonomic reactivity of the subjects was tested 1–4 weeks prior to HRCM in supine, sitting, standing, and walking positions. In pairwise tests, RSA decreased from supine to standing and walking, and from sitting to standing and walking (Figure 2A). RMSSD decreased from supine to sitting, standing and walking (Figure 2B). SI decreased significantly from supine to walking and from sitting to walking (Figure 2C). PEP did not show any significant change in response to posture change (Figure 2D). The heart rate changed significantly during each posture change (Figure 2E). In general, the parasympathetic activity showed a decrease and the sympathetic activity showed an increase in responses to changes in posture.

### Autonomic Reactivity Associated With Motor Patterns

A total of 145 eligible motor patterns from the 11 participants were included in the analysis. The motor patterns were comprised of 42 Motor Complexes; 28 HAPWs, 45 HAPW-SPWs, and 30 isolated SPWs. We first asked the question whether or not the occurrence of a motor pattern was associated with changes in HRV parameters, comparing activity in the 2 min before a motor pattern to the period in which the motor pattern took place using the Wilcoxon matched pair signed-rank test. Parasympathetic reactivity (both RSA and RMSSD) increased significantly (W-M); *p* < 0.0001) during the motor activity. Furthermore, both SI (W-M; *p* < 0.0001) and PEP (W-M; *p* < 0.001) showed a significant decrease in sympathetic reactivity during the motor activity. The heart rate did not change significantly during motor activity.

**FIGURE 3:**
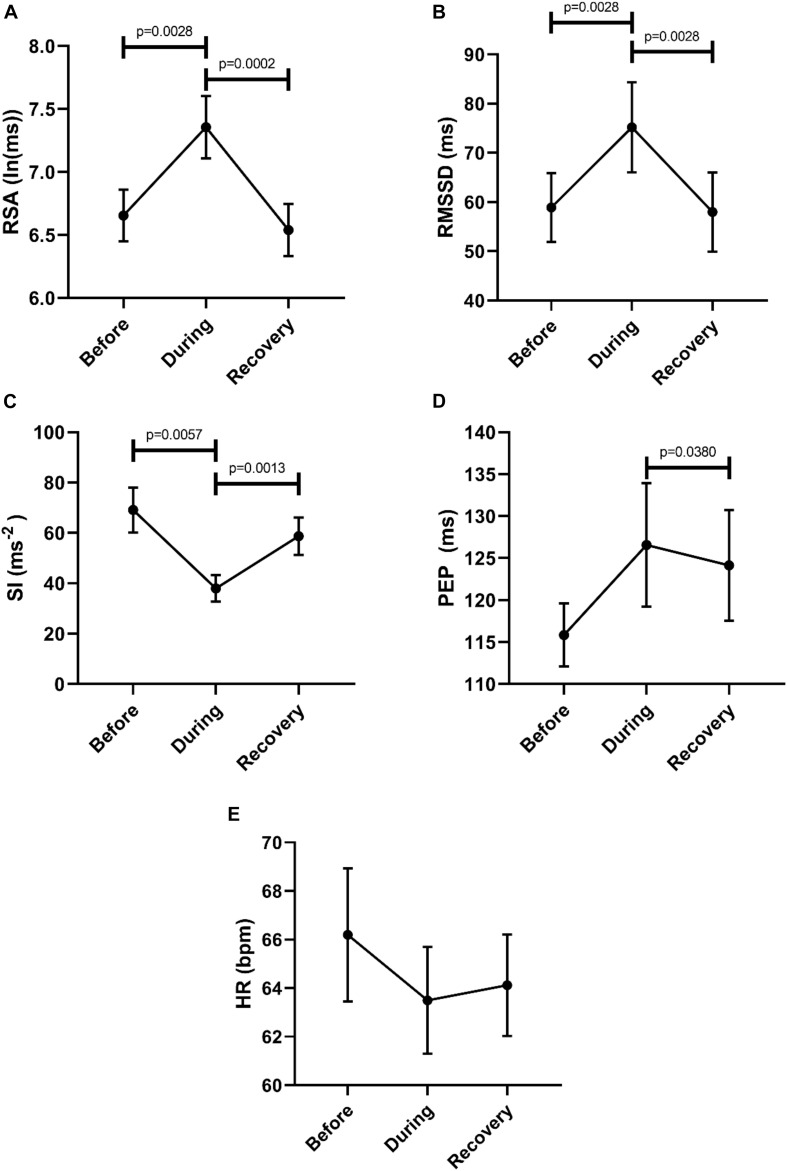
Changes in autonomic activity in response to all motor patterns. Data sets are derived by averaging all 145 motor patterns within subjects, hence *n* = 11. The Friedman test was applied for assessment of significance, followed by the Dunn’s multiple comparisons test. **(A)** RSA increased significantly during the motor activity and recovered within 2 min. **(B)** RMSSD increased significantly during the motor activity and recovered. **(C)** SI decreased significantly during the motor activity. **(D)** PEP decreased significantly after motor activity. **(E)** Heart rate remained unchanged.

Then we asked the question whether or not a recovery of autonomic activity to pre-motor pattern levels took place in the first 2 min after the motor pattern. Here we compared the 2 min before a motor pattern, the period during the motor pattern and the 2 min period after the motor pattern using the Freidman test followed by the Dunn’s multiple comparison test. The data shown in Figure 3 confirm that parasympathetic activity increased during a motor pattern compared to its baseline and then recovered during the 2 min following the motor pattern. According to the SI, sympathetic activity decreased during a motor pattern, and recovered in the next 2 min. For PEP, only the recovery phase showed a significant decline (increased sympathetic activity). Heart rate did not change significantly.

### Changes in Autonomic Reactivity Associated With the Different Motor Pattern Categories

#### Motor Complexes

Comparison of the 2 min quiet period preceding Motor Complexes with the period during the motor pattern revealed that this motor pattern was associated with an increase in parasympathetic activity reflected by an increase in RSA (W-M; *p* = 0.0039) and RMSSD (W-M; *p* = 0.0078) (*n* = 11). Motor Complexes were associated with a simultaneous decrease in sympathetic activity as reflected by an increase in the PEP value (W-M; *p* = 0.0078). During the Motor Complexes, the heart rate did not change.

**FIGURE 4:**
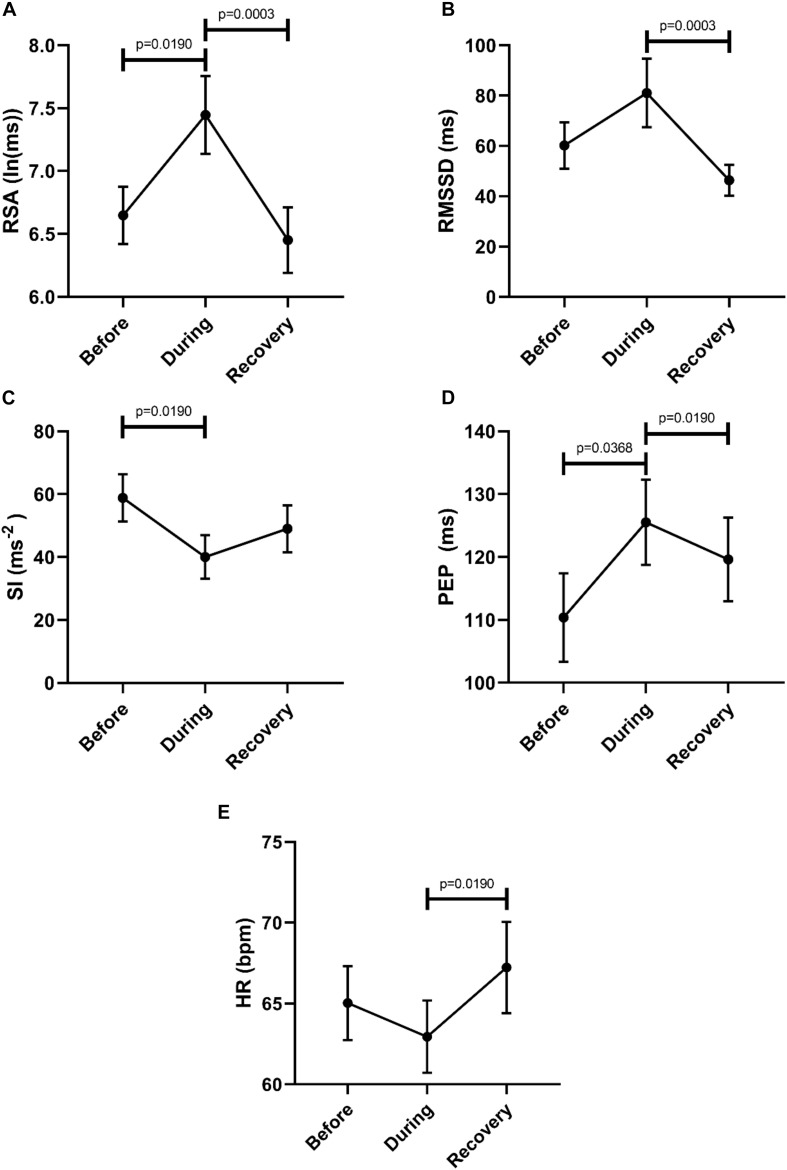
Overall changes in autonomic activity in response to Motor Complexes (*N* = 42; *n* = 9). The following HRV parameters were assessed: RSA **(A)**, RMSSD **(B)**, the Baevsky’s Stress Index (SI) **(C)**, PEP **(D)**, and heart rate **(E)**. Data sets were derived by averaging all motor patterns within subjects, hence *n* = 9). The Friedman test was applied for assessment of significance followed by Dunn’s multiple comparisons test (F-M).

In order to evaluate a potential recovery we analyzed autonomic parameters before, during and after the Motor Complexes. This confirmed an increase in parasympathetic activity reflected by an increase in RSA, and a decrease in sympathetic activity reflected by both SI and PEP (Figure 4). Recovery was significant for RSA, RMSSD and PEP (Figure 4). The heart rate was significantly higher in the recovery period compared to during the motor pattern (Figure 4).

**FIGURE 5:**
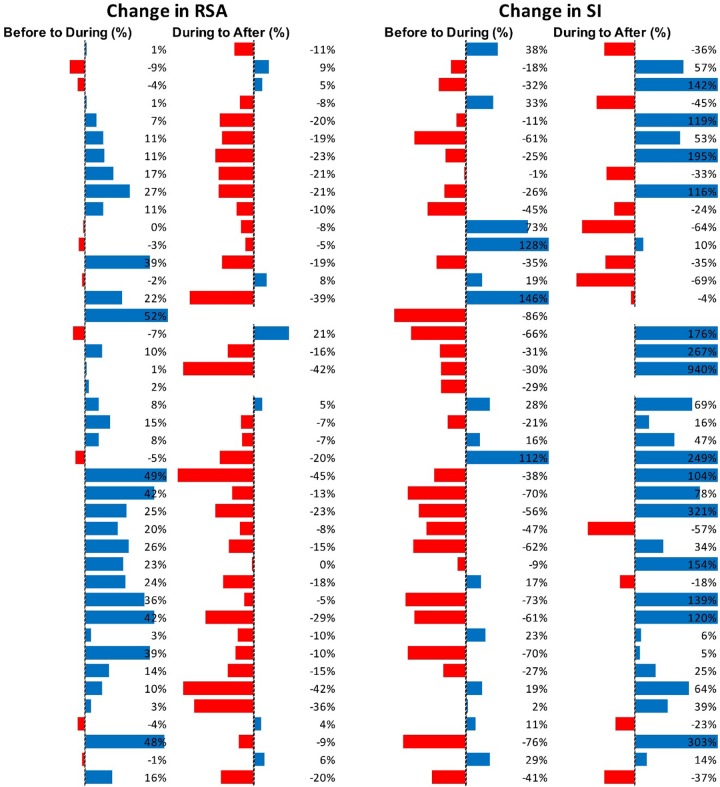
Changes in autonomic activity in response to all individual Motor Complexes (*N* = 42). Data are shown as% change. The Friedman test was applied for assessment of significance followed by Dunn’s multiple comparisons test. RSA: before to during *p* = 0.0007; during to recovery, *p* < 0.0001. SI: before to during *p* = 0.0104; during to recovery, *p* ≤ 0.0001 (F-D).

Figure 5 shows RSA and SI reactivity of all 42 Motor Complexes as percent change. RMSSD and PEP are shown in Supplementary Figure S1.

The analysis of the other motor patterns were executed in the same manner as the Motor Complexes.

#### HAPW-SPWs

Compared to the 2 min quiet period preceding the HAPW-SPWs, this motor pattern was associated with an increase in RSA (W-M; *p* = 0.0469) and a decrease in SI (*p* = 0.0313). Other parameters did not change significantly.

**FIGURE 6:**
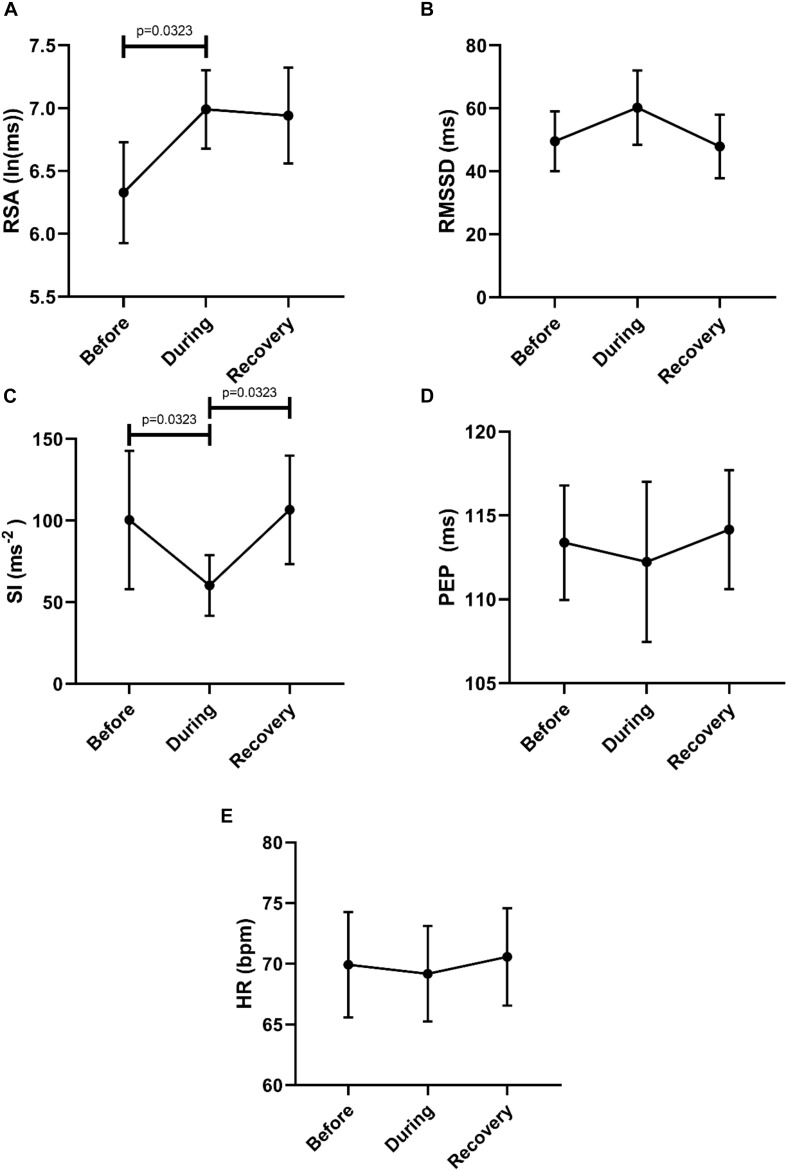
Overall changes in autonomic activity in response to HAPW-SPW’s. Data sets are derived by averaging all 45 motor patterns within 7 subjects, hence *n* = 7. The following HRV parameters were assessed: RSA **(A)**, RMSSD **(B)**, the Baevsky’s Stress Index (SI) **(C)**, PEP **(D)**, and heart rate **(E)**. The Friedman test was applied for assessment of significance followed by Dunn’s multiple comparisons test (F-D-M).

To assess potential recovery, the data sets before, during and after motor activity were compared (Figure 6). First, this confirmed an increase in parasympathetic activity (RSA) and a decrease in sympathetic activity (SI). Only the recovery after SI reached significance (Figure 6C). The heart rate did not change significantly.

**FIGURE 7:**
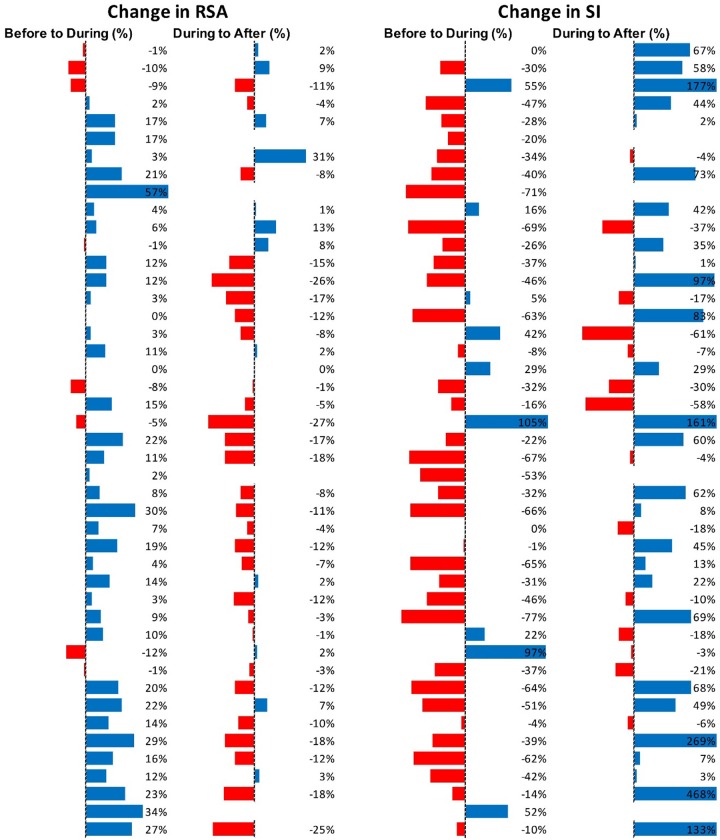
Changes in autonomic activity in response to all HAPW-SPW’s *N* = 45. Data are shown as % change. The Friedman test was applied for assessment of significance followed by Dunn’s multiple comparisons test. RSA: before to during *p* = 0.0018; during to recovery, *p* ≤ 0.0001. SI: before to during *p* = 0.0022; during to recovery, *p* < 0.0001 (F-D).

Figure 7 shows RSA and SI reactivity of all 45 HAPW-SPWs as percent change. RMSSD and PEP are shown in Supplementary Figure S2.

#### HAPWs

Comparing to the 2 min quiet period preceding the HAPWs, this motor pattern was associated with an increase in RSA (W-M; *p* = 0.0313) and a decrease in SI (W-M; *p* = 0.0313). Other parameters did not change significantly.

**FIGURE 8:**
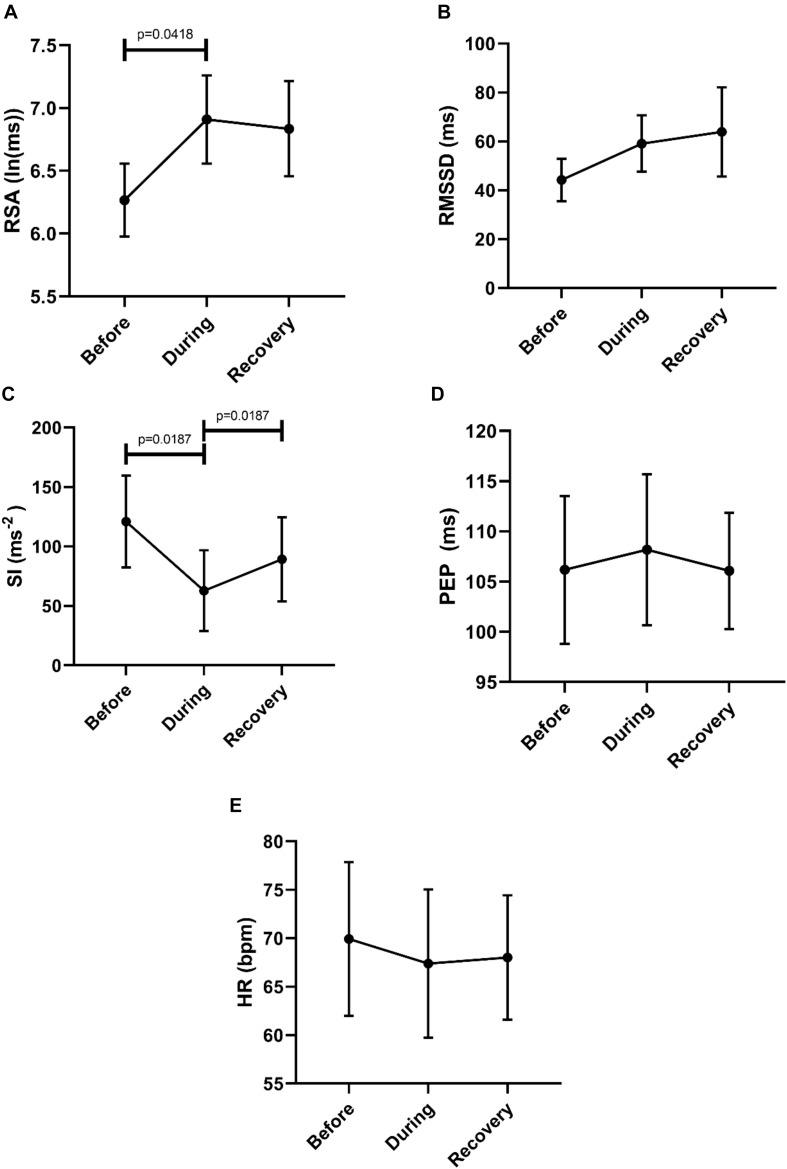
Overall changes in autonomic activity in response to HAPW’s. Data sets are derived by averaging all motor patterns within subjects, hence *n* = 6). The following HRV parameters were assessed: RSA **(A)**, RMSSD **(B)**, the Baevsky’s Stress Index (SI) **(C)**, PEP **(D)**, and heart rate **(E)**. The Friedman test was applied for assessment of significance followed by Dunn’s multiple comparisons test (F-D-M).

To assess potential recovery, the data sets before, during and after motor activity were compared, based on mean values from each individual (*n* = 6) (Figure 8). First, this confirmed that RSA increased significantly from before to during and that the SI decreased significantly comparing before to during. The SI recovered significantly within 2 min after the HAPW. Heart rate did not change.

**FIGURE 9:**
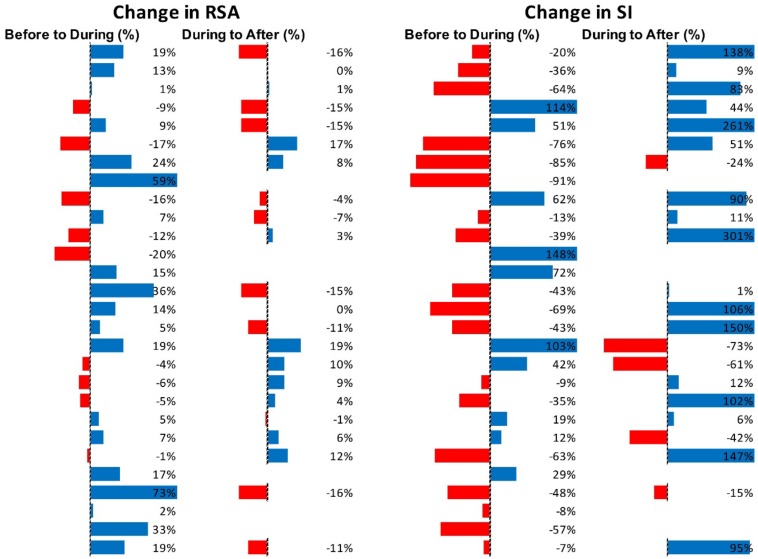
Changes in autonomic activity in response to all HAPW’s (*N* = 28). Data are shown as % change. The Friedman test was applied for assessment of significance followed by Dunn’s multiple comparisons test. RSA: before to during, not significant; during to recovery, not significant. SI: before to during *p* = 0.0389; during to recovery, *p* = 0.004 (F-D).

Figure 9 shows RSA and SI reactivity of all 28 HAPWs as percent change. RMSSD and PEP are shown in Supplementary Figure S3.

#### SPWs

Compared to the 2 min quiet period preceding the SPWs, this motor pattern was not associated with any significant changes in HRV parameters.

**FIGURE 10:**
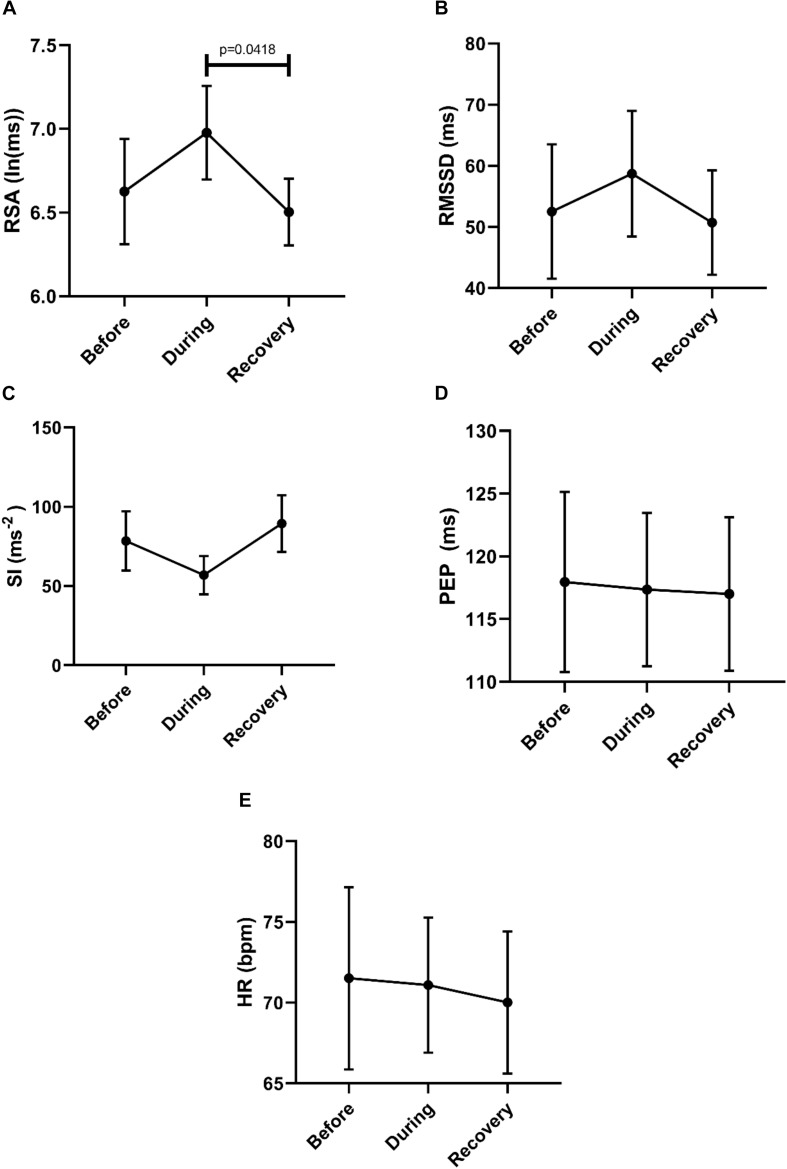
Overall Changes in autonomic activity in response to 30 SPW’s. Data sets were derived by averaging all motor patterns within subjects, hence *n* = 6. The following HRV parameters were assessed: RSA **(A)**, RMSSD **(B)**, the Baevsky’s Stress Index (SI) **(C)**, PEP **(D)**, and heart rate **(E)**. The Friedman test was applied for assessment of significance followed by Dunn’s multiple comparisons test (F-D-M).

To assess potential recovery, the data sets before, during and after motor activity were compared, based on mean values from each individual (*n* = 6) (Figure 10). Recovery of RSA was significant, but the differences between during and after did not reach significance in the first 2 min after the motor pattern for any of the other HRV parameters (F-D-M). The heart rate did not change significantly.

**FIGURE 11:**
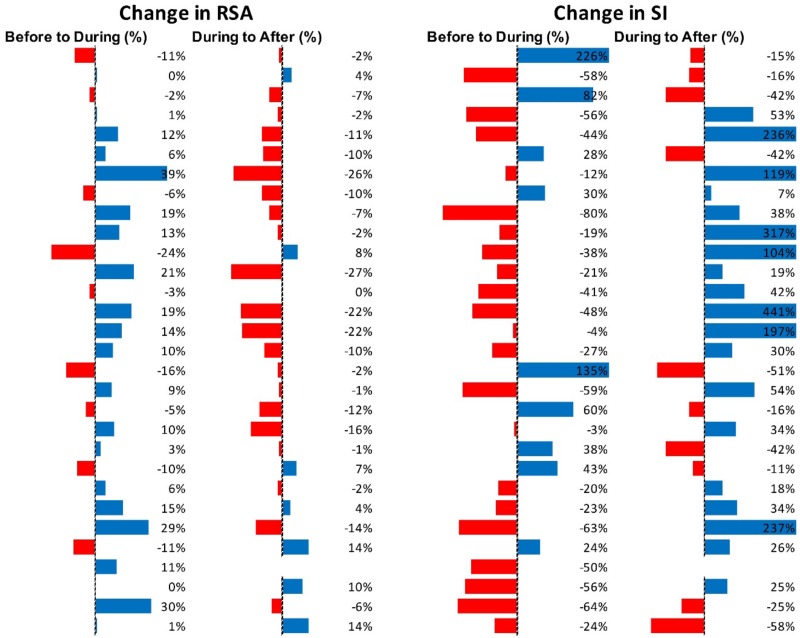
Changes in autonomic activity in response to all SPW’s (*N* = 30). Data are shown as % change. The Friedman test was applied for assessment of significance followed by Dunn’s multiple comparisons test. RSA: before to during, ns; during to recovery, *p* = 0.0142. SI: before to during: ns.; during to recovery, *p* = 0.0021 (F-D).

Figure 11 shows RSA and SI reactivity of all SPWs as percent change. RMSSD and PEP are shown in Supplementary Figure S4.

### Correlations Between General Autonomic Reactivity and Reactivity Associated With Motor Patterns

**FIGURE 12:**
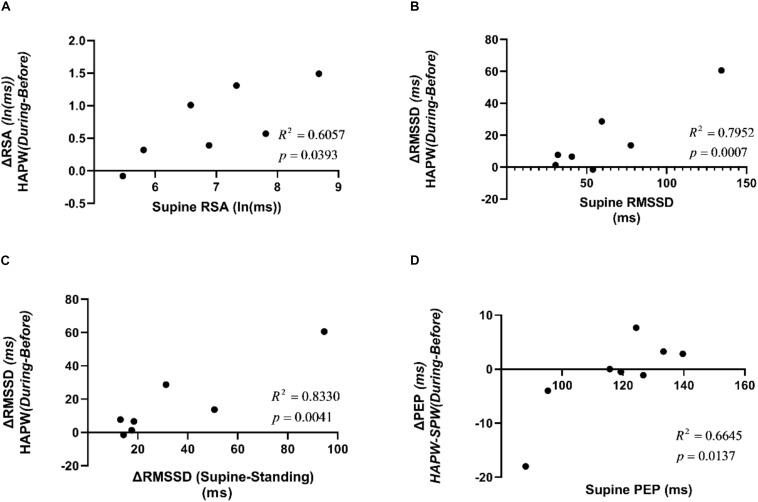
Correlations between supine HRV parameters and changes in HRV parameters due to posture changes compared to the changes in those measures due to motor activity. **(A)** Correlation between RSA during supine rest and change in RSA during HAPW. **(B)** Correlation between RMSSD during supine rest and change in RSA during HAPW. **(C)** Correlation between change in RMSSD from supine to standing and change in RSA during HAPW. **(D)** Correlation between PEP during supine rest and change in PEP during HAPW-SPW.

The HAPW associated change in RSA from before to during the motor pattern was correlated with the supine value of RSA (*R*^2^ = 0.6057, *p* < 0.05) (Figure 12A). The HAPW associated change was also correlated with the supine value of RMSSD (*R*^2^ = 0.7952, *p* < 0.05) as well as the change in RMSSD from supine to standing (*R*^2^ = 0.8330, *p* < 0.05) (Figures 12B,C). The supine value of PEP showed a correlation to the change in PEP value during HAPW-SPW (*R*^2^ = 0.6645, *p* < 0.05) (Figure 12D). The remaining measures of autonomic reactivity during supine as well as change from supine to standing were not significantly correlated to changes in autonomic activity during motor patterns (p > 0.05) (Supplementary Figures S5–S8).

## Discussion

Abnormal colonic motility, incontinence, and constipation including the inability to have spontaneous bowel movements may be associated with organic pathophysiology such as dysfunction of the enteric nervous system (Obermayr et al., 2013) or a marked reduction in ICC (Knowles and Farrugia, 2011). Alternatively, colonic motility may be abnormal due to dysfunctional brain-gut communication, such as an inability to sense a stimulus that would generate an urge to defecate, or an inability to properly respond to an urge to defecate. The ANS is involved in both sensation (a trigger for initiation of colonic motor patterns) and execution of motility and this is controlled or affected by the CAN (Roy and Green, 2019). Brain-gut communication dysfunction may also result in more subtle changes leading to hard stool or hyper-responsiveness to environmental stress (Chen et al., 2011; Mayer and Tillisch, 2011; Mazurak et al., 2012). The present study suggests some tools to investigate this further. A better understanding of the role of (abnormal) autonomic activity in conjunction with colonic motor dysfunction will lead to better understanding of underlying pathophysiology and may pave the way for evidence based treatment involving the autonomic control systems (Knowles et al., 2001; Knowles and Farrugia, 2011; Knowles et al., 2013; Bonaz et al., 2016).

The goal of this study was to assess whether autonomic activity associated with colonic motor patterns would be reflected in changes in HRV. Taking all motor patterns into account, it is clear that colonic motor patterns are accompanied by increased parasympathetic activity and decreased sympathetic activity, reflected in HRV changes, that may occur without eliciting a significant change in heart rate (Figure 3).

Motor Complexes, HAPW-SPWs and HAPWs are all associated with an increase in RSA and a decrease in SI. Hence RSA and SI may best reflect autonomic activity in the colon during these motor patterns as compared to RMSSD and PEP. It is important to state that SI and PEP do not measure identical sympathetic reactivity, SI is based on HRV whereas PEP is based on vascular tone changes.

The SPW, which is a very low amplitude pressure wave, did not significantly change the autonomic measures employed here.

Measurements of supine RSA and RSA changes due to changes in body position are deemed to be stable measures of general autonomic health (Ernst, 2017) and deemed relevant for assessment of ANS function in IBS (Jarrett et al., 2016; Polster et al., 2018). In the present study, the magnitudes of RSA and RMSSD change evoked by change in body position from supine to standing showed a marked decrease in parasympathetic reactivity, as expected (Mestanik et al., 2019). There was no significant change in PEP between supine and standing, which is consistent with the fact that moderate exercise may not show significant changes in sympathetic reactivity *as measured by PEP* (Alex et al., 2013). Nevertheless, SI showed a significant increase in sympathetic reactivity from supine or sitting to walking.

We show here that the magnitude of RSA and RMSSD changes evoked by change in body position from supine to standing was positively correlated with the magnitude of RSA and RMSSD changes associated with the HAPW. Hence, RSA or RMSSD reactivity to posture change may be a useful predictor of the ability to generate motor patterns that are dependent on parasympathetic activity. This may not be of clinical significance for healthy subjects, but if such correlations would be confirmed and/or other correlations were to be discovered in patients with colon motor dysfunction, general autonomic reactivity may have predictive value and/or develop into parameters that might be evaluated for improvement in pathophysiology in response to treatment.

The NTS is critical for regulation of both heart rate and gut motility (Browning and Travagli, 2014; Babic et al., 2015) involving distinct regions within the NTS; nevertheless, there is significant cross talk between neurons affecting heart rate and gut function (Castle et al., 2005). Indeed, functionally identified NTS neurons appear to lack viscerotopic organization despite the fact that many distinct reflex pathways for the gastrointestinal tract, the lung and the heart are routed through the NTS (Janig, 2006). The NTS may therefore be a nexus for interactions between autonomic regulation of the three systems. In addition to significant integration of inputs from all these areas within the NTS (Janig, 2006), there are direct projections to the NTS from the cortex, amygdala and the hypothalamus (van der Kooy et al., 1984). Hence specific activities from higher brain centers likely influence both cardiac and gut regulation through the NTS (Castle et al., 2005; Roy and Green, 2019). We hypothesize a model in which the autonomic changes observed in the present study are associated with the following actions: motor patterns associated with rectal balloon distention or rectal bisacodyl start with rectal stimulation leading to sensory neural activity entering the defecation center in the sacral cord followed by sensory neural activity going up the spinal cord to Barrington’s nucleus and the NTS (Taché et al., 2004, 2005; Grundy et al., 2006). The Barrington nucleus has control over the sacral parasympathetic nucleus and thus has control over the motility of the bladder and the distal colon (Valentino et al., 1999; Sasaki and Sato, 2013). Multi-directional communication between the NTS, the vagal motor nucleus and higher brain center follows (van der Kooy et al., 1984). Parasympathetic vagal and sacral motor activity then initiate colonic motor patterns that start in the proximal colon and proceed all the way to the rectum ending with relaxation of the anal sphincter (Chen et al., 2018). Although the vagal motor nucleus is predominantly associated with proximal colon activity, and hence is likely involved in triggering motor patterns that start in the proximal colon, it may well be that vagal fibers are controlling motor patterns all the way to the rectum (Powley, 2000). Spontaneous propulsive motor patterns may use similar pathways. We propose that it is this neural activity within the extrinsic ANS which accompanies initiation and execution of motor patterns, that is reflected in changes in HRV.

The preejection period or PEP is the time elapsed between the electrical depolarization of the left ventricle (Q in the QRS complex in the ECG) and the beginning of ventricular ejection and represents the period of left ventricular contraction with the cardiac valves closed (Lanfranchi et al., 2017); it is an index of cardiac sympathetic (β-adrenergic) activity (Krohová et al., 2017). The SI is purely based on the RR intervals. With an increase in sympathetic activity, the HRV decreases, hence the variation in RR intervals tends to decrease (Baevsky and Chernikova, 2017). Although PEP and the SI are based on different metrics, both showed responsivity to motor patterns elicited during manometry, in the expected directions; but it is likely that not all HRV parameters reflect non-cardiac autonomic activity equally (Massaro and Pecchia, 2019). Only the Motor Complexes were associated with a change in PEP. in contrast, the Baevsky index was markedly affected during all motor patterns except the SPW, suggesting that the Baevsky index better reflects changes in sympathetic activity related to colon motor patterns.

Although the number of participants was relatively small, a large number of motor patterns (145) was available for analysis. Strong arguments have been put forward for the relevance of studies where a large number of observations are made on a relatively small number of experimental participants (Smith and Little, 2018). The temporal relationship between motor patterns and changes in autonomic measures of HRV demonstrates that colonic motor patterns are accompanied by significant parasympathetic autonomic neural activity and by withdrawal of sympathetic activity. Sacral nerves to the colon contain sensory and motor neurons, hence the autonomic activity may reflect motor neuron activity that initiates the motor patterns and/or sensory neural activity that precedes the motor patterns or is induced by the motor patterns. The distinction between the influences of sensory factors versus motor activities needs more stringent investigation. In the present study, motor patterns were initiated by different stimuli such as meal and bisacodyl; in the future, when more data become available, it will be important and interesting to subdivide the motor patterns according to their particular initiators. It is possible that motor patterns such as the SPW are associated with parasympathetic autonomic changes but that these changes do not exceed the magnitude of the inevitable physiologic “noise” in the recordings.

In conclusion, colonic motor patterns are associated with activity in the ANS that is reflected in HRV parameters. These autonomic measures may serve as proxies for autonomic neural dysfunction in patients with colonic dysmotility.

## Data Availability Statement

All datasets generated for this study are included in the article/Supplementary Material.

## Ethics Statement

The studies involving human participants were reviewed and approved by the Hamilton Integrated Research Ethics Board. The participants provided their written informed consent to participate in this study.

## Author Contributions

All studies were conducted at the McMaster University Medical Center. J-HC performed the HRCM studies. J-HC and JH developed HRCM at McMaster University. YY and MA executed the HRV analyses assisted by MF, KS, NM, and LL. MA made substantial contributions to the Baevsky index implementation. WT, KZ, NM, and LL took part in execution and data analysis of HRV. SP was instrumental in the design of HRCM and HRV analysis. KM and LS were critical for HRV analysis and statistical analysis. ER and DA were instrumental in execution of HRCM. JH supervised all analyses. All authors were involved in discussing and revising the manuscript.

## Conflict of Interest

The authors declare that the research was conducted in the absence of any commercial or financial relationships that could be construed as a potential conflict of interest.

## Abbreviations

ANS: Autonomic Nervous System
CAN: Central Autonomic Network
ECG: Electrocardiogram
HAPW: High Amplitude Propagating Pressure Wave
HAPW-SPW: High Amplitude Propagating Pressure Wave followed by a Simultaneous Pressure Wave
HRCM: High-resolution Colonic Manometry
HRV: Heart Rate Variability
NTS: Nucleus Tractus Solitarius
PEP: Pre-Ejection Period
RSA: Respiratory Sinus Arrhythmia
SI: Baevsky Stress Index
SPW: Simultaneous Pressure Wave.

## References

[B1] Alex C. Lindgren M. Shapiro P. A. McKinley P. S. Brondolo E. N. Myers M. M. (2013). Aerobic exercise and strength training effects on cardiovascular sympathetic function in healthy adults: a randomized controlled trial. *Psychosom. Med.* 75 375–381. 10.1097/psy.0b013e3182906810 23630307 PMC4518731

[B2] Babic T. Ambler J. Browning K. N. Travagli R. A. (2015). Characterization of synapses in the rat subnucleus centralis of the nucleus tractus solitarius. *J. Neurophysiol.* 113 466–474. 10.1152/jn.00598.2014 25355962 PMC4297788

[B3] Baevsky R. M. Chernikova A. G. (2017). Heart rate variability analysis: physiological foundations and main methods. *Cardiometry* 66–67. 10.12710/cardiometry.2017.10.6676

[B4] Beauchaine T. P. Thayer J. F. (2015). Heart rate variability as a transdiagnostic biomarker of psychopathology. *Int. J. Psychophysiol.* 98 338–350. 10.1016/j.ijpsycho.2015.08.004 26272488

[B5] Bharucha A. E. (2012). High amplitude propagated contractions. *Neurogastroenterol. Motil.* 24 977–982. 10.1111/nmo.12019 23057554 PMC3471560

[B6] Bharucha A. E. Camilleri M. Low P. A. Zinsmeister A. R. (1993). Autonomic dysfunction in gastrointestinal motility disorders. *Gut* 34 397–401. 10.1136/gut.34.3.397 8472990 PMC1374149

[B7] Bharucha A. E. Low P. A. Camilleri M. Burton D. Gehrking T. L. Zinsmeister A. R. (2008). Pilot study of pyridostigmine in constipated patients with autonomic neuropathy. *Clin. Auton. Res.* 18 194–202. 10.1007/s10286-008-0476-x 18622640 PMC2536749

[B8] Bonaz B. Sinniger V. Pellissier S. (2016). Vagal tone: effects on sensitivity, motility, and inflammation. *Neurogastroenterol. Motil.* 28 455–462. 10.1111/nmo.12817 27010234

[B9] Brierley S. M. Jones R. C. Gebhart G. F. Blackshaw L. A. (2004). Splanchnic and pelvic mechanosensory afferents signal different qualities of colonic stimuli in mice. *Gastroenterology* 127 166–178. 10.1053/j.gastro.2004.04.008 15236183

[B10] Brookes S. Chen N. Humenick A. Spencer N. J. Costa M. (2016). Extrinsic sensory innervation of the gut: structure and function. *Adv. Exp. Med. Biol.* 891 63–69. 10.1007/978-3-319-27592-5_7 27379635

[B11] Browning K. N. Travagli R. A. (2014). Central nervous system control of gastrointestinal motility and secretion and modulation of gastrointestinal functions. *Compr. Physiol.* 4 1339–1368. 10.1002/cphy.c130055 25428846 PMC4858318

[B12] Callaghan B. Furness J. B. Pustovit R. V. (2018). Neural pathways for colorectal control, relevance to spinal cord injury and treatment: a narrative review. *Spinal Cord* 56 199–205. 10.1038/s41393-017-0026-2 29142293

[B13] Castle M. Comoli E. Loewy A. D. (2005). Autonomic brainstem nuclei are linked to the hippocampus. *Neuroscience* 134 657–669. 10.1016/j.neuroscience.2005.04.031 15975727

[B14] Chen J. H. Parsons S. P. Shokrollahi M. Wan A. Vincent A. D. Yuan Y. (2018). Characterization of simultaneous pressure waves as biomarkers for colonic motility assessed by high-resolution colonic manometry. *Front. Physiol. Gastrointest. Sci.* 9:1248. 10.3389/fphys.2018.01248 30294277 PMC6159752

[B15] Chen J. H. Yu Y. Yang Z. Yu W. Z. Chen W. L. Kim M. J. M. (2017). Intraluminal pressure patterns in the human colon assessed by high-resolution manometry. *Sci. Rep.* 7:41436. 10.1038/srep41436 28216670 PMC5316981

[B16] Chen J. Y. Blankstein U. Diamant N. E. Davis K. D. (2011). White matter abnormalities in irritable bowel syndrome and relation to individual factors. *Brain Res.* 1392 121–131. 10.1016/j.brainres.2011.03.069 21466788

[B17] Costa M. Brookes S. H. (2008). Architecture of enteric neural circuits involved in intestinal motility. *Eur. Rev. Med. Pharmacol. Sci.* 12(Suppl. 1), 3–19. 18924440

[B18] Devroede G. Lamarche J. (1974). Functional importance of extrinsic parasympathetic innervation to the distal colon and rectum in man. *Gastroenterology* 66 273–280. 10.1016/s0016-5085(74)80114-9 4810918

[B19] Draghici A. E. Taylor J. A. (2016). The physiological basis and measurement of heart rate variability in humans. *J. physiol. anthropol.* 35:22. 27680542 10.1186/s40101-016-0113-7 PMC5039876

[B20] Ernst G. (2017). Heart-rate variability-more than heart beats. *Front. Public Health* 5:240. 10.3389/fpubh.2017.00240 28955705 PMC5600971

[B21] Fazeli M. S. Collet J. P. MacNeily A. E. Afshar K. (2016). Cardiac autonomic nervous system activity in children with bladder and bowel dysfunction. *J. Urol.* 195 1245–1249. 10.1016/j.juro.2015.11.020 26926551

[B22] Furness J. B. (2012). The enteric nervous system and neurogastroenterology. *Nat. Rev. Gastroenterol. Hepatol.* 9 286–294. 10.1038/nrgastro.2012.32 22392290

[B23] Ghasemi A. Zahediasl S. (2012). Normality tests for statistical analysis: a guide for non-statisticians. *Int. J. Endocrinol. Metab.* 10 486–489. 10.5812/ijem.3505 23843808 PMC3693611

[B24] Grundy D. Al-Chaer E. D. Aziz Q. Collins S. M. Ke M. Tache Y. (2006). Fundamentals of neurogastroenterology: basic science. *Gastroenterology* 130 1391–1411. 10.1053/j.gastro.2005.11.060 16678554

[B25] Huizinga J. D. (2018). “The physiology and pathophysiology of interstitial cells of cajal: pacemaking, innervation, and stretch sensation,” in *Physiology of the Gastrointestinal Tract*, eds Said H. Kaunitz J. K. Ghishan F. Merchant J. Wood J., (Amsterdam: Elsevier), 305–336.

[B26] Janig W. (2006). *The Integrative Action of the Autonomic Nervous System: Neurobiology of Homeostasis*. Cambridge, MA: Cambridge University Press.

[B27] Jarrett M. E. Cain K. C. Barney P. G. Burr R. L. Naliboff B. D. Shulman R. (2016). Balance of autonomic nervous system predicts who benefits from a self-management intervention program for irritable bowel syndrome. *J. Neurogastroenterol. Motil.* 22 102–111. 10.5056/jnm15067 26459461 PMC4699727

[B28] Jáuregui-Renaud K. Hermosillo A. G. Márquez M. F. Ramos-Aguilar F. Hernández-Goribar M. Cárdenas M. (2001). Repeatability of heart rate variability during simple cardiovascular reflex tests on healthy subjects. *Arch. Med. Res.* 32 21–26. 10.1016/s0188-4409(00)00255-1 11282175

[B29] Knowles C. H. Farrugia G. (2011). Gastrointestinal neuromuscular pathology in chronic constipation. *Best Pract. Res. Clin. Gastroenterol.* 25 43–57. 10.1016/j.bpg.2010.12.001 21382578 PMC4175481

[B30] Knowles C. H. Lindberg G. Panza E. De Giorgio R. (2013). New perspectives in the diagnosis and management of enteric neuropathies. *Nat. Rev. Gastroenterol. Hepatol.* 10 206–218. 10.1038/nrgastro.2013.18 23399525

[B31] Knowles C. H. Scott S. M. Lunniss P. J. (2001). Slow transit constipation: a disorder of pelvic autonomic nerves? *Dig. Dis. Sci.* 46 389–401. 11281190 10.1023/a:1005665218647

[B32] Krohová J. Czippelová B. Turianiková Z. Lazarová Z. Tonhajzerová I. Javorka M. (2017). Preejection period as a sympathetic activity index: a role of confounding factors. *Physiol. Res.* 66 S265–S275. 28937241 10.33549/physiolres.933682

[B33] Lanfranchi P. A. Pepin J. L. Somers V. K. (2017). “Cardiovascular physiology: autonomic control in health and in sleep disorders,” in *Principles and Practice of Sleep Medicine*, eds Kryger M. Roth T. Dement W. C., (Philadelphia, PA: Elsevier), 142–154.

[B34] Massaro S. Pecchia L. (2019). Heart rate variability (HRV) analysis: a methodology for organizational neuroscience. *Organ. Res. Methods* 22 354–393. 10.1177/1094428116681072

[B35] Mathewson K. J. Van Lieshout R. J. Saigal S. Boyle M. H. Schmidt L. A. (2014). Reduced respiratory sinus arrhythmia in adults born at extremely low birth weight: evidence of premature parasympathetic decline. *Int. J. Psychophysiol.* 93 198–203. 10.1016/j.ijpsycho.2014.04.005 24747727 PMC4114728

[B36] Mayer E. A. Tillisch K. (2011). The brain-gut axis in abdominal pain syndromes. *Annu. Rev. Med.* 62 381–396. 10.1146/annurev-med-012309-103958 21090962 PMC3817711

[B37] Mazurak N. Seredyuk N. Sauer H. Teufel M. Enck P. (2012). Heart rate variability in the irritable bowel syndrome: a review of the literature. *Neurogastroenterol. Motil.* 24 206–216. 10.1111/j.1365-2982.2011.01866.x 22256893

[B38] McIntyre A. S. Thompson D. G. (1992). Adrenergic control of motor and secretory function in the gastrointestinal tract. *Aliment. pharmacol. ther.* 6 125–142. 10.1111/j.1365-2036.1992.tb00257.x 1600036

[B39] Mestanik M. Mestanikova A. Langer P. Grendar M. Jurko A. Sekaninova N. (2019). Respiratory sinus arrhythmia–testing the method of choice for evaluation of cardiovagal regulation. *Respir. physiol. neurobiol.* 259 86–92. 10.1016/j.resp.2018.08.002 30086386

[B40] Obermayr F. Hotta R. Enomoto H. Young H. M. (2013). Development and developmental disorders of the enteric nervous system. *Nat. Rev. Gastroenterol. Hepatol.* 10 43–57. 10.1038/nrgastro.2012.234 23229326

[B41] Parati G. Di Rienzo M. (2003). Determinants of heart rate and heart rate variability. *J. Hypertens.* 21 477–480. 10.1097/00004872-200303000-00007 12640235

[B42] Polster A. Friberg P. Gunterberg V. Öhman L. Le Nevé B. Törnblom H. (2018). Heart rate variability characteristics of patients with irritable bowel syndrome and associations with symptoms. *Neurogastroenterol. Motil.* 30:e13320. 10.1111/nmo.13320 29575352

[B43] Powley T. L. (2000). Vagal input to the enteric nervous system. *Gut* 47(Suppl. 4), iv30–iv32. discussion iv36, 11076904 10.1136/gut.47.suppl_4.iv30 PMC1766799

[B44] Quigley K. S. Stifter C. A. (2006). A comparative validation of sympathetic reactivity in children and adults. *Psychophysiology* 43 357–365. 10.1111/j.1469-8986.2006.00405.x 16916431

[B45] Roy H. A. Green A. L. (2019). The central autonomic network and regulation of bladder function. *Front. neurosci.* 13:535. 10.3389/fnins.2019.00535 31263396 PMC6585191

[B46] Sasaki M. Sato H. (2013). Polysynaptic connections between Barrington’s nucleus and sacral preganglionic neurons. *Neurosci. Res.* 75 150–156. 10.1016/j.neures.2012.11.008 23257509

[B47] Schmidt L. A. Santesso D. L. Miskovic V. Mathewson K. J. McCabe R. E. Antony M. M. (2012). Test-retest reliability of regional electroencephalogram (EEG) and cardiovascular measures in social anxiety disorder (SAD). *Int. J. Psychophysiol.* 84 65–73. 10.1016/j.ijpsycho.2012.01.011 22280842

[B48] Shaffer F. Ginsberg J. P. (2017). An overview of heart rate variability metrics and norms. *Front. Public Health* 5:258. 10.3389/fpubh.2017.00258 29034226 PMC5624990

[B49] Smith P. L. Little D. R. (2018). Small is beautiful: in defense of the small-N design. *Psychon. bull. rev.* 25 2083–2101. 10.3758/s13423-018-1451-8 29557067 PMC6267527

[B50] Spencer N. J. Dinning P. G. Brookes S. J. Costa M. (2016). Insights into the mechanisms underlying colonic motor patterns. *J. Physiol.* 594 4099–4116. 10.1113/JP271919 26990133 PMC4967752

[B51] Szurszewski J. H. Ermilov L. G. Miller S. M. (2002). Prevertebral ganglia and intestinofugal afferent neurones. *Gut* 51(Suppl. 1), i6–i10. 10.1136/gut.51.suppl_1.i6 12077055 PMC1867710

[B52] Tache Y. (2003). “The parasympathetic nervous system in the pathophysiology of the gastrointestinal tract,” in *Handbook of the Autonomic Nervous System*, eds Bolis C. L. Licinio J. Govoni S., (Basel: Marcel Dekker Inc.), 463–504.

[B53] Taché Y. Martinez V. Wang L. Million M. (2004). CRF1 receptor signaling pathways are involved in stress-related alterations of colonic function and viscerosensitivity: implications for irritable bowel syndrome. *Br. J. Pharmacol.* 141 1321–1330. 10.1038/sj.bjp.0705760 15100165 PMC1574904

[B54] Taché Y. Million M. Nelson A. G. Lamy C. Wang L. (2005). Role of corticotropin-releasing factor pathways in stress-related alterations of colonic motor function and viscerosensibility in female rodents. *Gend. Med.* 2 146–154. 10.1016/s1550-8579(05)80043-9 16290887

[B55] Valentino R. J. Miselis R. R. Pavcovich L. A. (1999). Pontine regulation of pelvic viscera: pharmacological target for pelvic visceral dysfunctions. *Trends Pharmacol. Sci.* 20 253–260. 10.1016/s0165-6147(99)01332-2 10366869

[B56] van der Kooy D. Koda L. Y. McGinty J. F. Gerfen C. R. Bloom F. E. (1984). The organization of projections from the cortex, amygdala, and hypothalamus to the nucleus of the solitary tract in rat. *J. Comp. Neurol.* 224 1–24. 10.1002/cne.902240102 6715573

[B57] Wang L. Martínez V. Larauche M. Taché Y. (2009). Proximal colon distension induces fos expression in oxytocin-, vasopressin-, CRF- and catecholamines-containing neurons in rat brain. *Brain Res.* 1247 79–91. 10.1016/j.brainres.2008.09.094 18955037 PMC3210201

