# Supplementary material for: Associations Between Colonic Motor Patterns and Autonomic Nervous System Activity Assessed by High-Resolution Manometry and Concurrent Heart Rate Variability
Source: Front Neurosci. 2020 Jan 23;13:1447. doi: 10.3389/fnins.2019.01447 (PMC6989554; doi:10.3389/fnins.2019.01447)
Supplement: Supplementary file 1 [file Data_Sheet_1.PDF]

SUPPLEMENTARY FIGURES

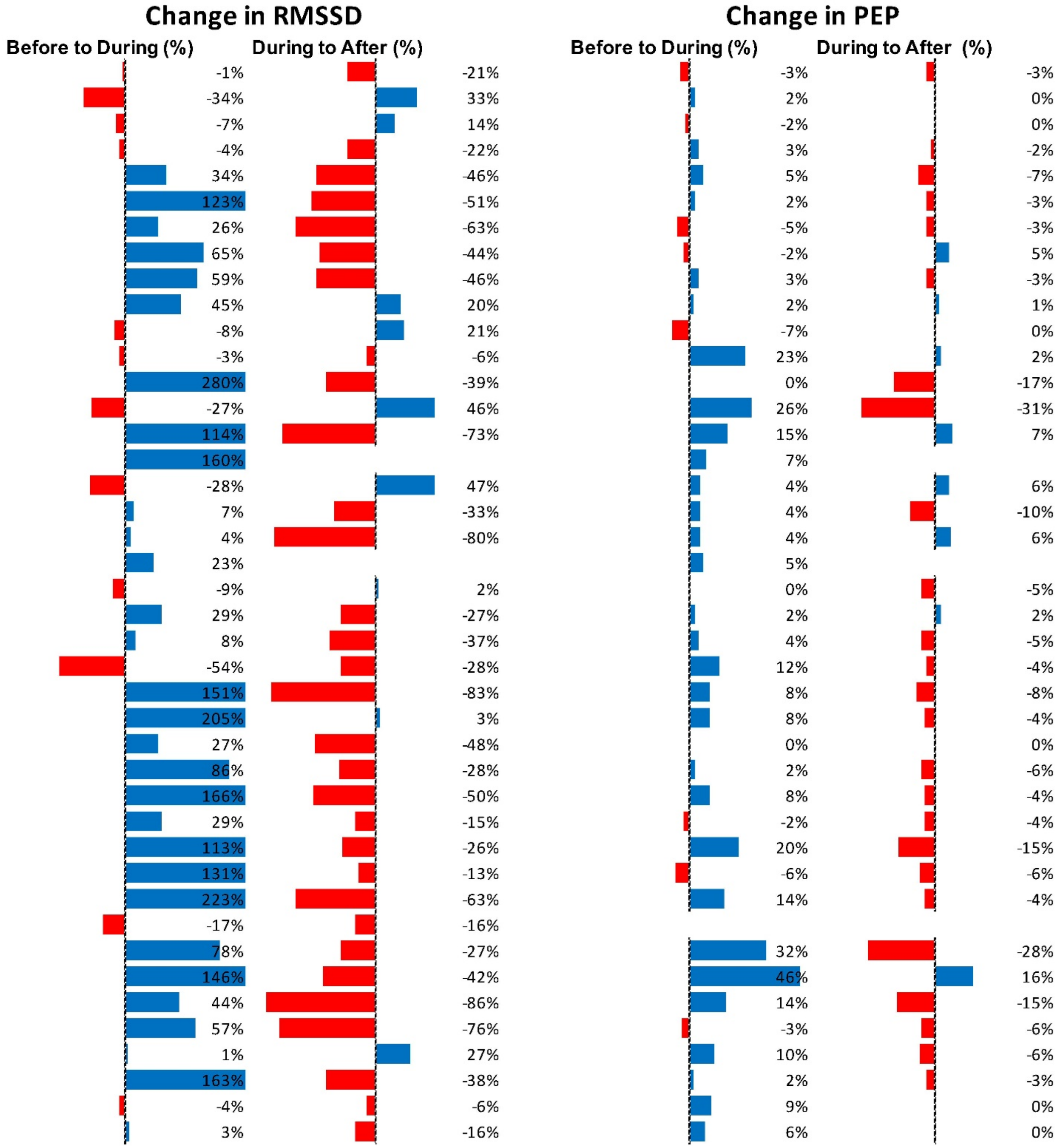

Figure S1: Changes in autonomic activity in response to all individual Motor Complexes. (N=42). Data are shown as % change. The Friedman test was applied for assessment of significance followed by Dunn's multiple comparisons test. RMSSD: before to during p=0.0104 ; during to recovery, P< 0.0001. PEP: before to during p=0.0250 ; during to recovery not significant (F-D)

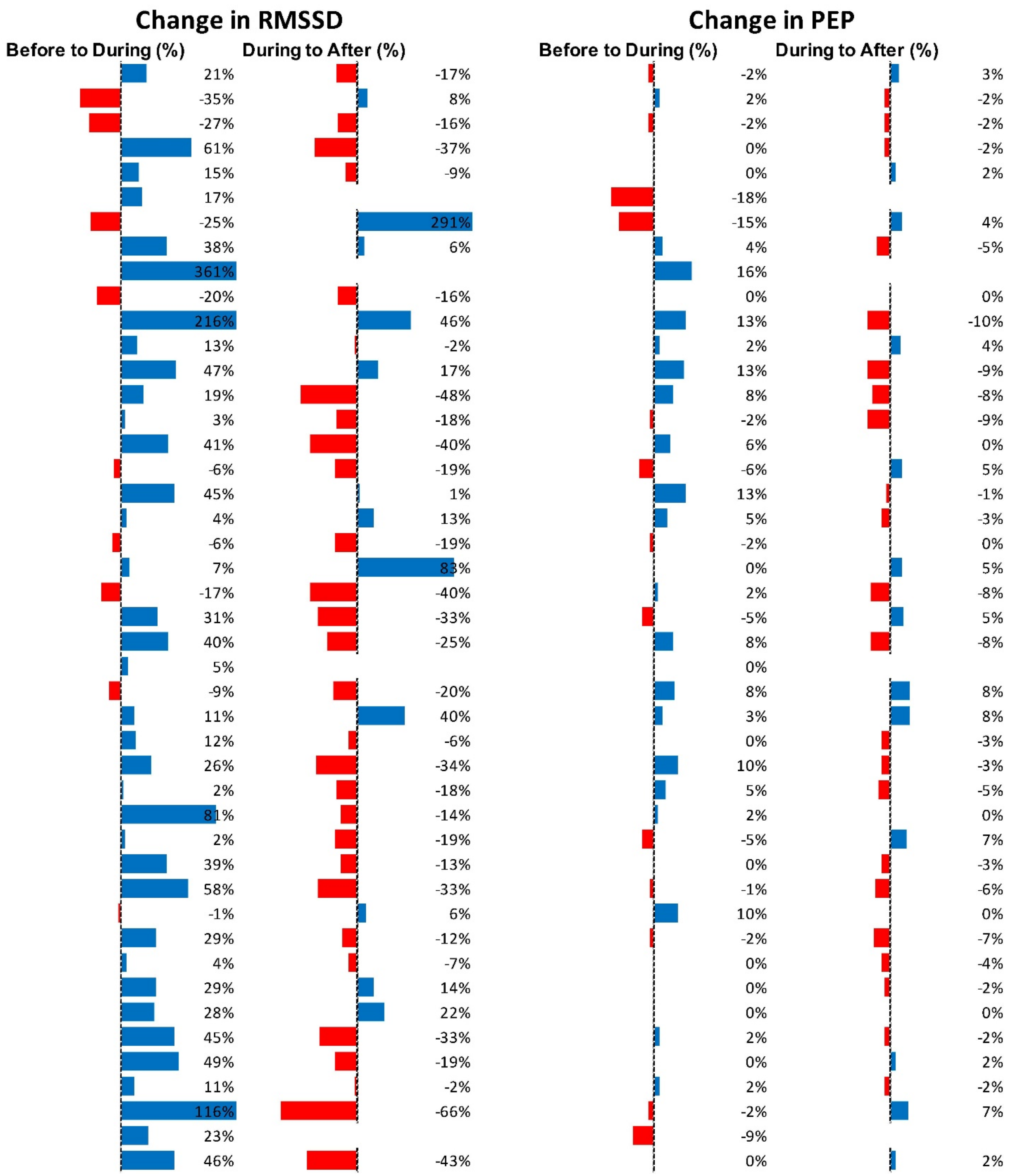

Figure S2: Changes in autonomic activity in response to all HAPW-SPW's

(N=45). Data are shown as % change. The Friedman test was applied for assessment of significance followed by Dunn's multiple comparisons test. RMSSD: before to during p=0.0001 ; during to recovery, p=<0.0001. PEP: before to during, not significant; during to recovery, not significant (F-D)

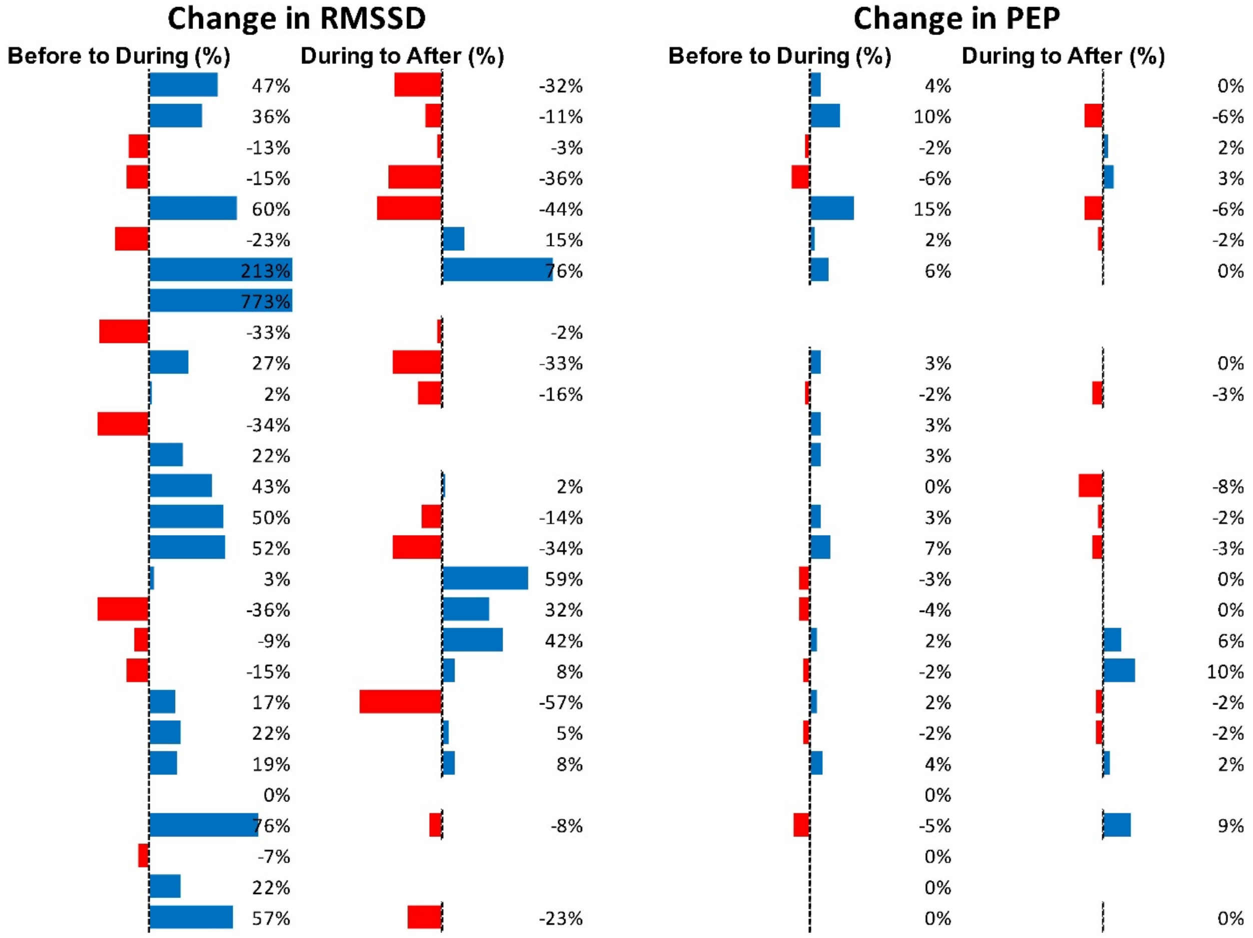

Figure S3: Changes in autonomic activity in response to all HAPW's (N=28). Data are shown as % change. The Friedman test was applied for assessment of significance followed by Dunn's multiple comparisons test. RMSSD: before to during, not significant; during to recovery, not significant. PEP: before to during, not significant. ; during to recovery, not significant (F-D)

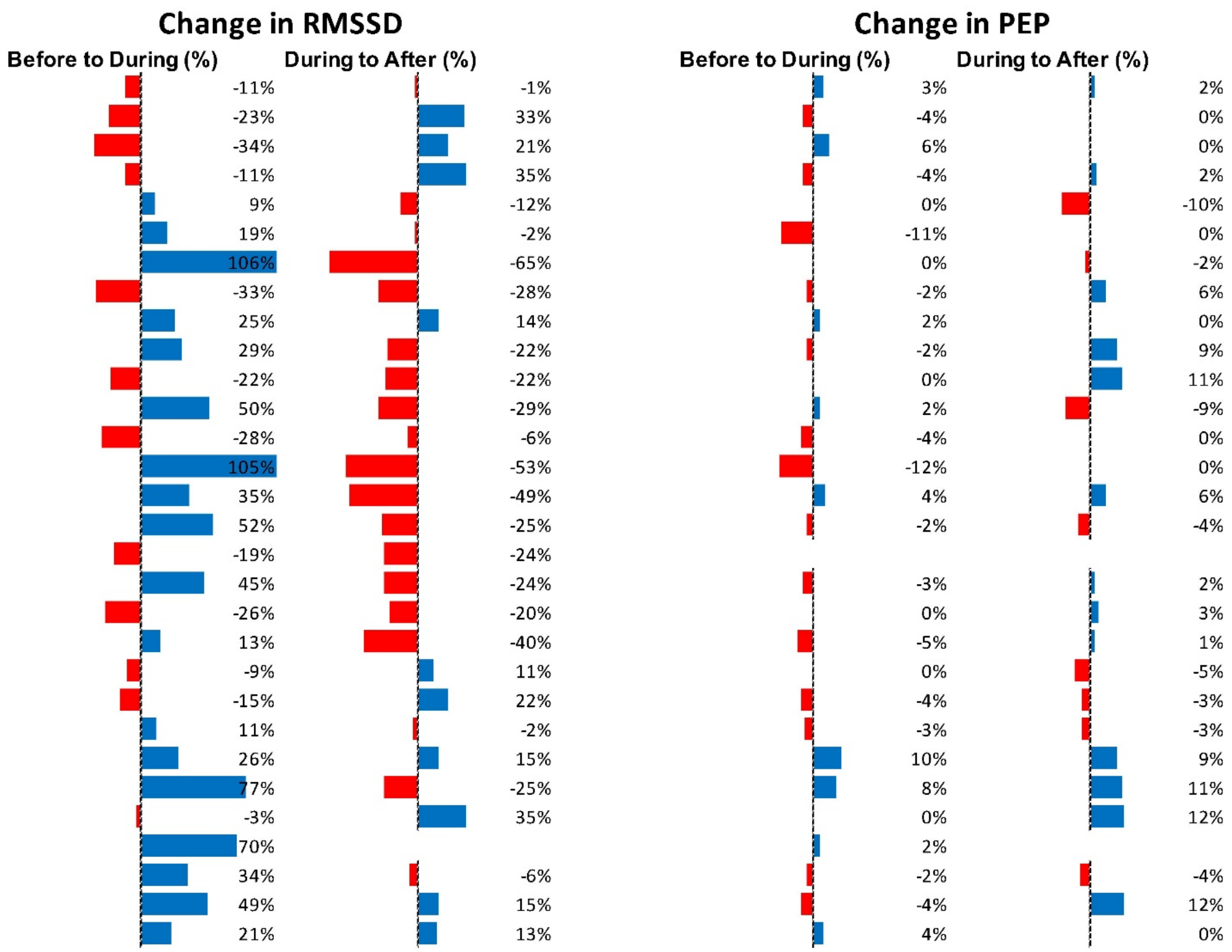

Figure S4: Changes in autonomic activity in response to all SPW's.

(N=30). Data are shown as % change. The Friedman test was applied for assessment of significance followed by Dunn's multiple comparisons test. RMSSD: before to during, not significant ; during to recovery, not significant. PEP: before to during: not significant. ; during to recovery, p=0.0151 (F-D)

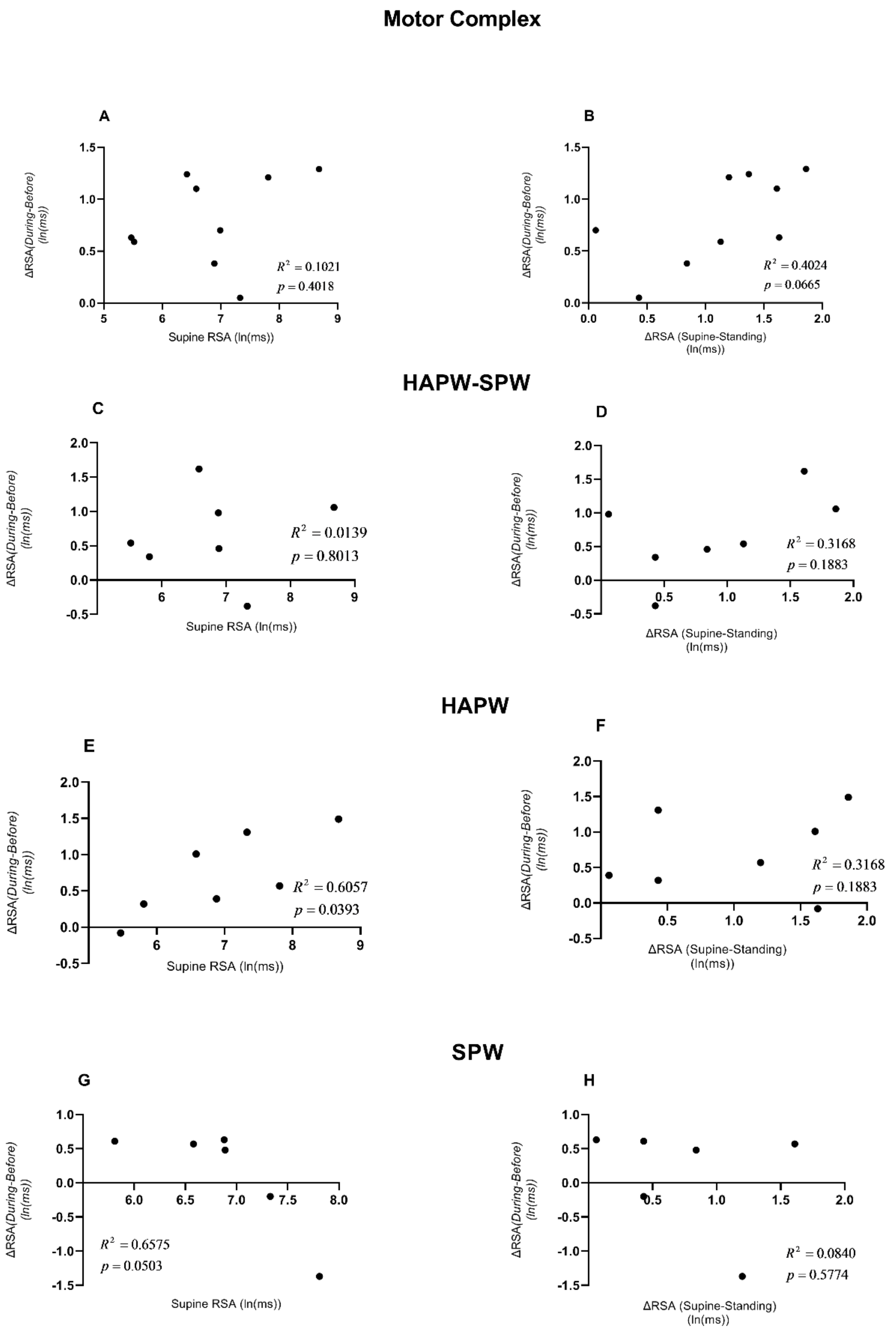

Motor Complex
A
ΔRSA(During-Before) (ln(ms))
Supine RSA (ln(ms))
$R^2 = 0.1021$
$p = 0.4018$
B
ΔRSA(During-Before) (ln(ms))
ΔRSA (Supine-Standing) (ln(ms))
$R^2 = 0.4024$
$p = 0.0665$
HAPW-SPW
C
ΔRSA(During-Before) (ln(ms))
Supine RSA (ln(ms))
$R^2 = 0.0139$
$p = 0.8013$
D
ΔRSA(During-Before) (ln(ms))
ΔRSA (Supine-Standing) (ln(ms))
$R^2 = 0.3168$
$p = 0.1883$
HAPW
E
ΔRSA(During-Before) (ln(ms))
Supine RSA (ln(ms))
$R^2 = 0.6057$
$p = 0.0393$
F
ΔRSA(During-Before) (ln(ms))
ΔRSA (Supine-Standing) (ln(ms))
$R^2 = 0.3168$
$p = 0.1883$
SPW
G
ΔRSA(During-Before) (ln(ms))
Supine RSA (ln(ms))
$R^2 = 0.6575$
$p = 0.0503$
H
ΔRSA(During-Before) (ln(ms))
ΔRSA (Supine-Standing) (ln(ms))
$R^2 = 0.0840$
$p = 0.5774$

Figure S5: Correlations between supine RSA and changes in RSA due to posture changes compared to the changes in RSA due to motor activity.

A. Correlation between supine RSA and change in RSA during Motor Complex
B. Correlation between change in RSA from supine to standing and change in RSA during Motor Complex
C. Correlation between supine RSA and change in RSA during HAPW-SPW's
D. Correlation between change in RSA from supine to standing and change in RSA during HAPW-SPW's
E. Correlation between supine RSA and change in RSA during HAPW's
F. Correlation between change in RSA from supine to standing and change in RSA during HAPW's
G. Correlation between supine RSA and change in RSA during SPW's
H. Correlation between change in RSA from supine to standing and change in RSA during SPW's

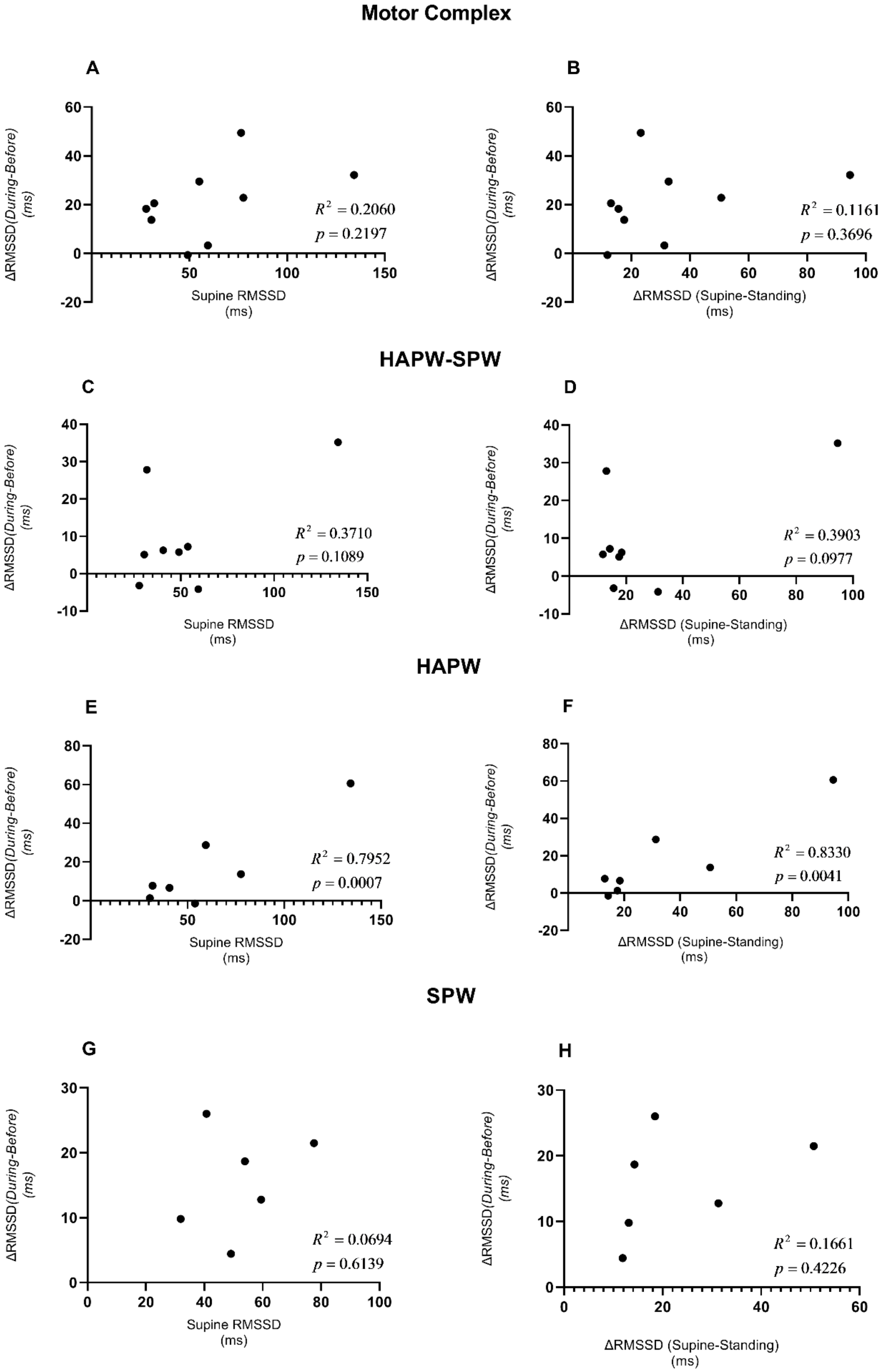
Motor Complex
A
ΔRMSSD(During-Before) (ms)
Supine RMSSD (ms)
$R^2 = 0.2060$
$p = 0.2197$
B
ΔRMSSD(During-Before) (ms)
ΔRMSSD (Supine-Standing) (ms)
$R^2 = 0.1161$
$p = 0.3696$
HAPW-SPW
C
ΔRMSSD(During-Before) (ms)
Supine RMSSD (ms)
$R^2 = 0.3710$
$p = 0.1089$
D
ΔRMSSD(During-Before) (ms)
ΔRMSSD (Supine-Standing) (ms)
$R^2 = 0.3903$
$p = 0.0977$
HAPW
E
ΔRMSSD(During-Before) (ms)
Supine RMSSD (ms)
$R^2 = 0.7952$
$p = 0.0007$
F
ΔRMSSD(During-Before) (ms)
ΔRMSSD (Supine-Standing) (ms)
$R^2 = 0.8330$
$p = 0.0041$
SPW
G
ΔRMSSD(During-Before) (ms)
Supine RMSSD (ms)
$R^2 = 0.0694$
$p = 0.6139$
H
ΔRMSSD(During-Before) (ms)
ΔRMSSD (Supine-Standing) (ms)
$R^2 = 0.1661$
$p = 0.4226$

Figure S6: Correlations between supine RMSSD and changes in RMSSD due to posture changes compared to the changes in RMSSD due to motor activity.

A. Correlation between supine RMSSD and change in RMSSD during Motor Complex
B. Correlation between change in RMSSD from supine to standing and change in RMSSD during Motor Complex
C. Correlation between supine RMSSD and change in RMSSD during HAPW-SPW's
D. Correlation between change in RMSSD from supine to standing and change in RMSSD during HAPW-SPW's
E. Correlation between supine RMSSD and change in RMSSD during HAPW's
F. Correlation between change in RMSSD from supine to standing and change in RMSSD during HAPW's
G. Correlation between supine RMSSD and change in RMSSD during SPW's
H. Correlation between change in RMSSD from supine to standing and change in RMSSD during SPW's

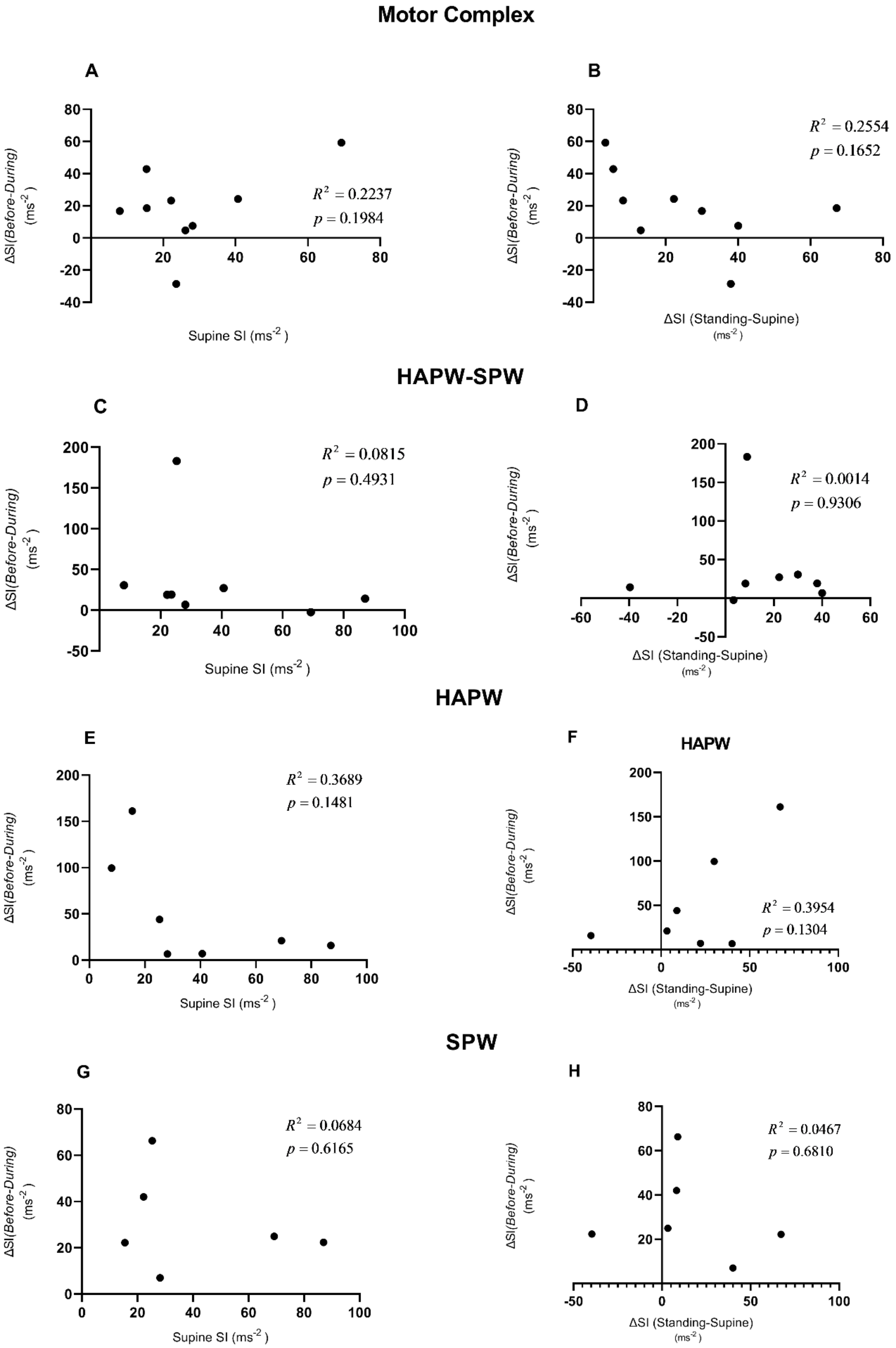
Motor Complex
A
ΔSI(Before-During) (ms^{-2})
Supine SI (ms^{-2})
$R^2 = 0.2237$
$p = 0.1984$
B
ΔSI (Standing-Supine) (ms^{-2})
$R^2 = 0.2554$
$p = 0.1652$
HAPW-SPW
C
$R^2 = 0.0815$
$p = 0.4931$
D
$R^2 = 0.0014$
$p = 0.9306$
HAPW
E
$R^2 = 0.3689$
$p = 0.1481$
F
HAPW
$R^2 = 0.3954$
$p = 0.1304$
SPW
G
$R^2 = 0.0684$
$p = 0.6165$
H
$R^2 = 0.0467$
$p = 0.6810$

Figure S7: Correlations between supine SI and changes in SI due to posture changes compared to the changes in SI due to motor activity.

A. Correlation between supine SI and change in SI during Motor Complex
B. Correlation between change in SI from supine to standing and change in SI during Motor Complex
C. Correlation between supine SI and change in SI during HAPW-SPW's
D. Correlation between change in SI from supine to standing and change in SI during HAPW-SPW's
E. Correlation between supine SI and change in SI during HAPW's
F. Correlation between change in SI from supine to standing and change in SI during HAPW's
G. Correlation between supine SI and change in SI during SPW's
H. Correlation between change in SI from supine to standing and change in SI during SPW's

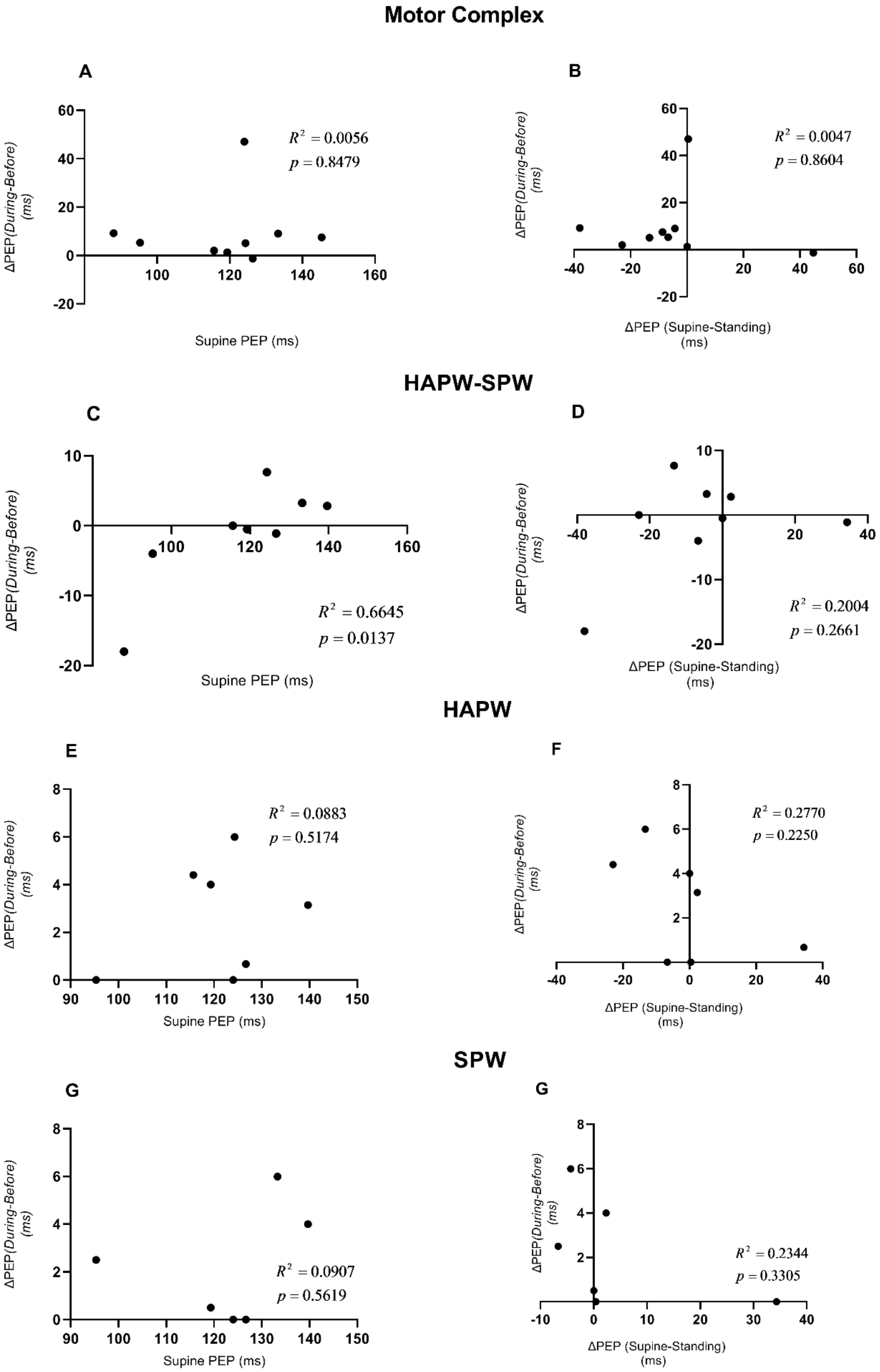
Motor Complex
A
ΔPEP(During-Before) (ms)
Supine PEP (ms)
$R^2 = 0.0056$
$p = 0.8479$
B
ΔPEP(During-Before) (ms)
ΔPEP (Supine-Standing) (ms)
$R^2 = 0.0047$
$p = 0.8604$
HAPW-SPW
C
ΔPEP(During-Before) (ms)
Supine PEP (ms)
$R^2 = 0.6645$
$p = 0.0137$
D
ΔPEP(During-Before) (ms)
ΔPEP (Supine-Standing) (ms)
$R^2 = 0.2004$
$p = 0.2661$
HAPW
E
ΔPEP(During-Before) (ms)
Supine PEP (ms)
$R^2 = 0.0883$
$p = 0.5174$
F
ΔPEP(During-Before) (ms)
ΔPEP (Supine-Standing) (ms)
$R^2 = 0.2770$
$p = 0.2250$
SPW
G
ΔPEP(During-Before) (ms)
Supine PEP (ms)
$R^2 = 0.0907$
$p = 0.5619$
G
ΔPEP(During-Before) (ms)
ΔPEP (Supine-Standing) (ms)
$R^2 = 0.2344$
$p = 0.3305$

Figure S8: Correlations between supine PEP and changes in PEP due to posture changes compared to the changes in PEP due to motor activity.

A. Correlation between supine PEP and change in PEP during Motor Complex
B. Correlation between change in PEP from supine to standing and change in PEP during Motor Complex
C. Correlation between supine PEP and change in PEP during HAPW-SPW's
D. Correlation between change in PEP from supine to standing and change in PEP during HAPW-SPW's
E. Correlation between supine PEP and change in PEP during HAPW's
F. Correlation between change in PEP from supine to standing and change in PEP during HAPW's
G. Correlation between supine PEP and change in PEP during SPW's
H. Correlation between change in PEP from supine to standing and change in PEP during SPW's
